# Supplementary material for: Diffusion-based deep learning method for augmenting ultrastructural imaging and volume electron microscopy
Source: Nat Commun. 2024 Jun 1;15:4677. doi: 10.1038/s41467-024-49125-z (PMC11144272; doi:10.1038/s41467-024-49125-z)
Supplement: Supplementary file 1 — Supplementary Information [file 41467_2024_49125_MOESM1_ESM.pdf]

Supplementary Information

Diffusion-based deep learning method for augmenting ultrastructural imaging and volume electron microscopy

Chixiang Lu[1], Kai Chen[1,2], Heng Qiu[1], Xiaojun Chen[2], Gu Chen[1], Xiaojuan Qi[3,†], Haibo Jiang[1,†]

From the [1]Department of Chemistry, The University of Hong Kong, Hong Kong, China; [2]School of Molecular Sciences, The University of Western Australia, Perth, Australia; [3]Department of Electrical and Electronic Engineering, The University of Hong Kong, Hong Kong, China

[†]Correspondence: Haibo Jiang, hbjiang@hku.hk and Xiaojuan Qi, xjqi@eee.hku.hk

Supplementary Figures

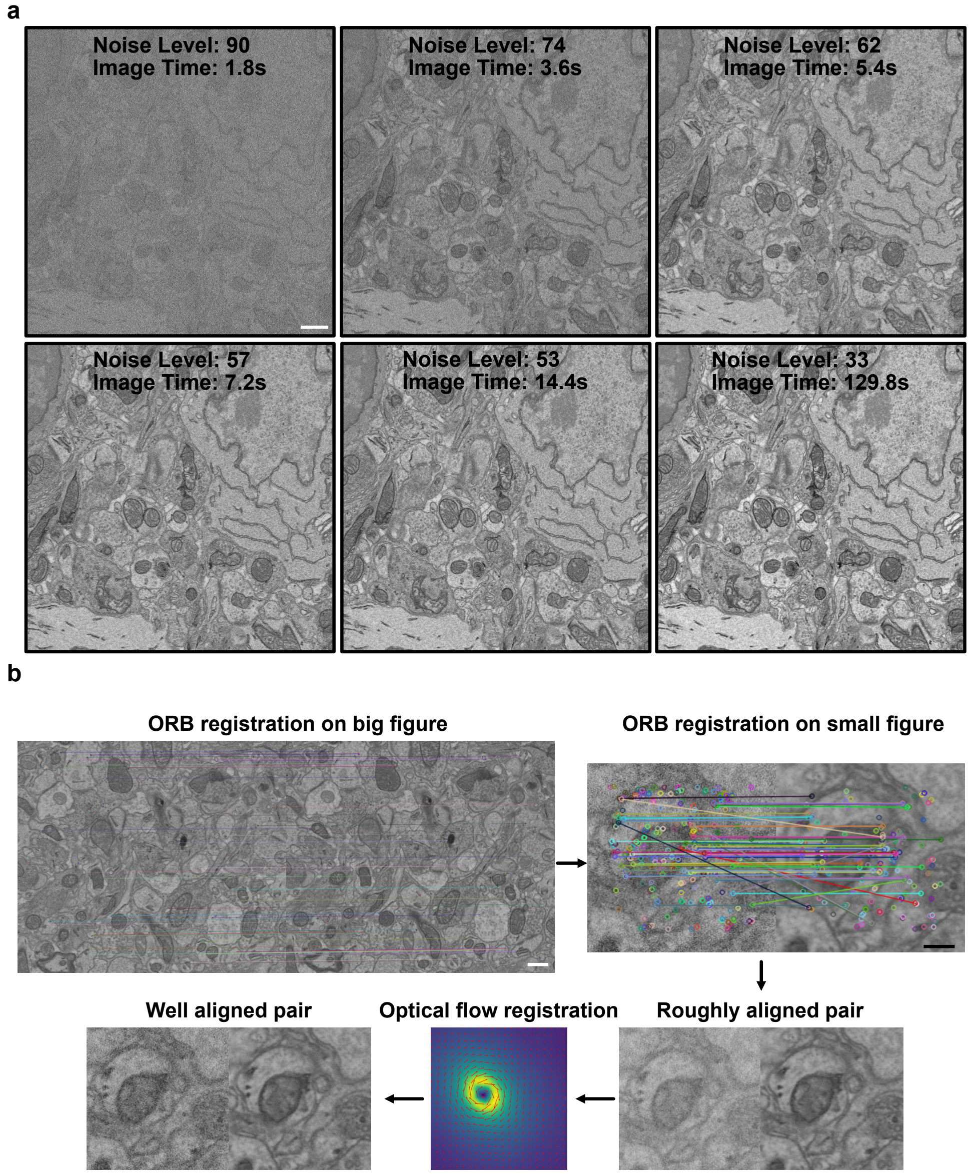

Supplementary Fig. 1 Denoise dataset acquisition and processing.

a. Data acquisition. A dataset of electron microscopy images with a 3.3 nm pixel size of the mouse brain cortex was acquired to train EMDiffuse. Various dwell times, ranging from 0.5 μs to 36 μs, representing different noise levels, are obtained to train a noise level-agnostic model and assesses the fidelity and limitation of EMDiffuse.

b. Data processing. ORB registration is employed for the alignment of images of various noise levels. An additional optical flow registration is incorporated to enhance the alignment of training samples to mitigate the drift and distortion during the EM imaging for effective model training. Scale bar, white, 0.5 μm; black, 0.1 μm.

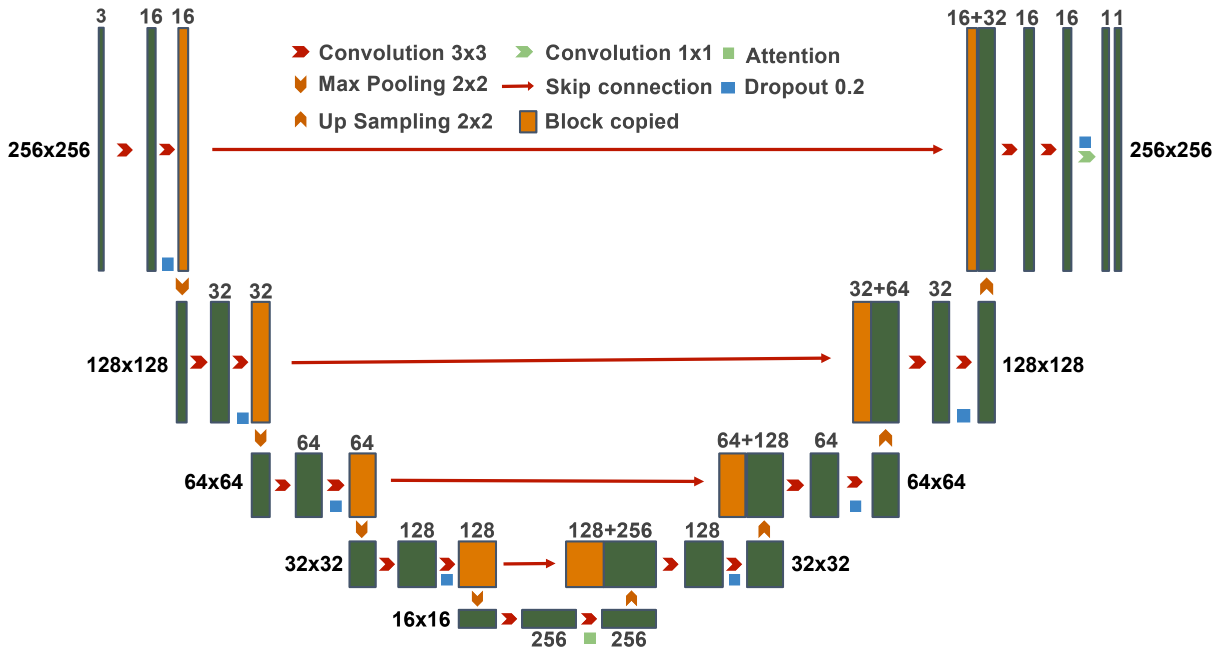

Supplementary Fig. 2 Network architecture of the UDiM model.
A traditional U-shape model comprising an encoder and a decoder is employed in every step of the diffusion chain. Attention is added to the bottleneck of the model to improve model performance.

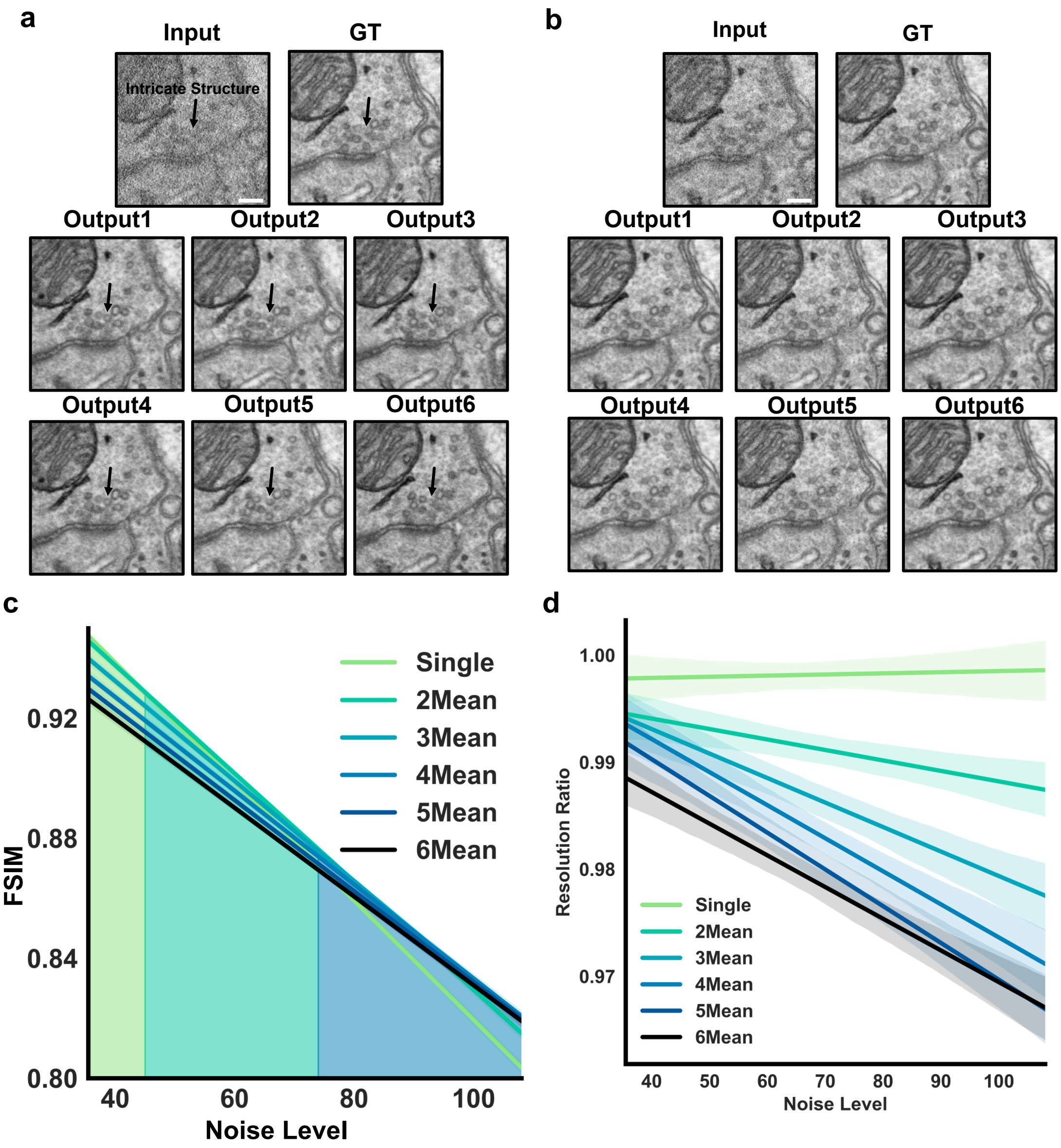

Supplementary Fig. 3 EMDiffuse samples one plausible solution at each test time, and the prediction can be optimized using the means of different outputs.

a, b. EMDiffuse samples one plausible solution from distribution. In cases where the intricate structure is heavily dominated by noise (a), variance exists in sampled images (*arrows*). For less noisy cases (b), outputs are highly consistent. Multiple outputs can be sampled, analyzed individually, or averaged to improve the final prediction. Scale bar, 0.1 µm.

c. Assessments of EMDiffuse performance based on FSIM. Shown are the FSIM of EMDiffuse-processed images of different noise levels and the mean of different numbers of outputs. The number of outputs for generating the mean result that yields the highest FSIM is selected for EMDiffuse prediction.

d. Quantification of resolution ratio (resolution of GT/resolution of EMDiffuse prediction) from the mean of different numbers of EMDiffuse outputs and different input noise levels. Scale bar, 0.1 µm.

Source data are provided as a Source Data file.

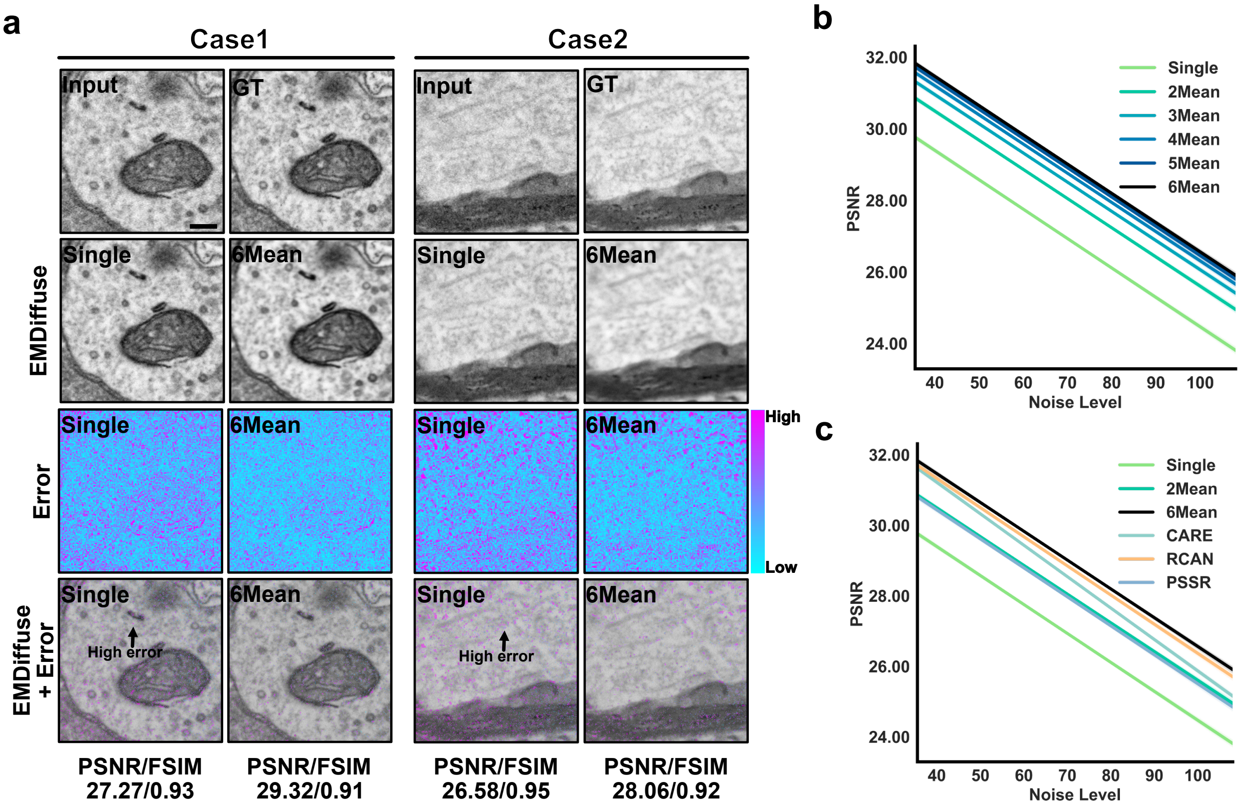

Supplementary Fig. 4 PSNR is impacted by random noise in ground-truth image.
a. PSNR measures the pixel-wise intensity similarity. The first row is the low noise level input and GT. The second row is the EMDiffuse prediction with the mean of six outputs (6mean) and without mean (single). Error maps (third row) and blend images of predictions and error maps (fourth row) indicate that the PSNR score is significantly affected by noise and does not correlate with structural similarity. Scale bar, 0.1 µm.
b. In the denoising task, the PSNR value rises with the increasing number of outputs used to calculate the mean prediction. The average of several outputs converges the minimum mean square error of solution distribution and, therefore, alleviates the impact of noise and raises the PSNR score.
c. Comparison of EMDiffuse with other methods in the denoising task. EMDiffuse outperforms all other methods in terms of PSNR with a mean calculation of six outputs, but the results are over-smoothed and the resolution is compromised.
Source data are provided as a Source Data file.

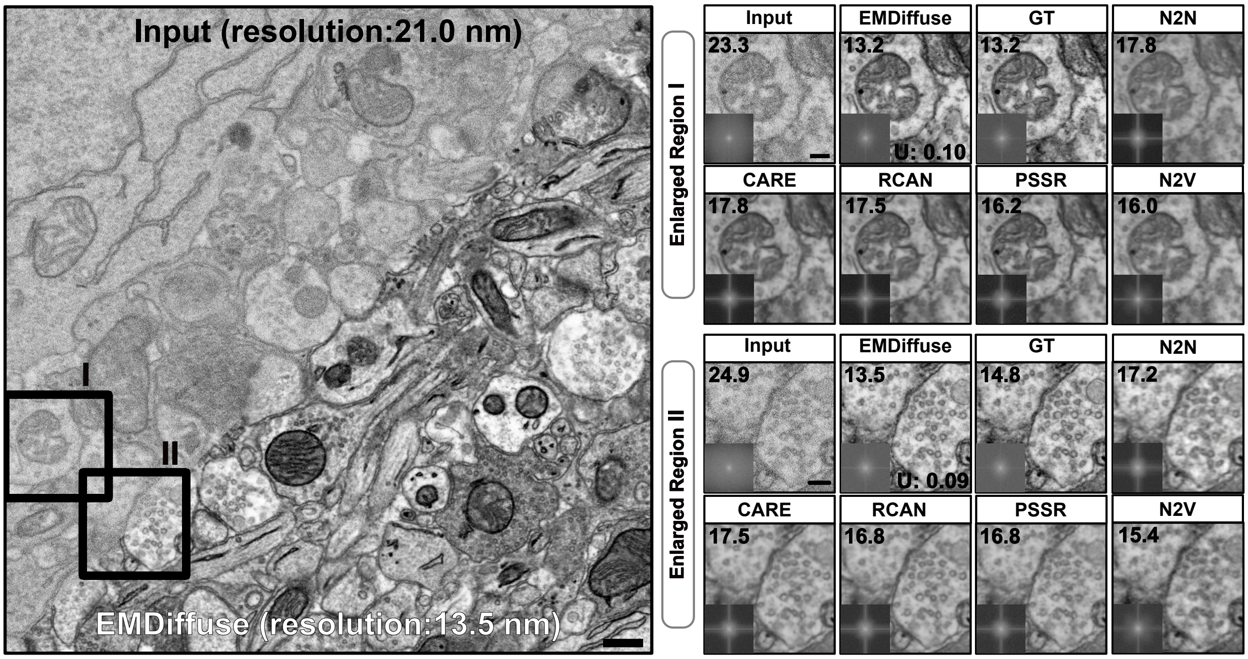

Supplementary Fig. 5 Additional examples of EMDiffuse-n for denoising EM images and comparison with other denoising methods.

Additional examples of denoising of mouse cortex EM images compared to other denoising methods. Left: a denoising example containing both the noisy input and the EMDiffuse-n prediction. Right: two enlarged regions of raw input, ground truth (GT), and predictions from EMDiffuse, CARE, RCAN, PSSR, Noise2Noise (N2N), and Noise2Void (N2V) denoising methods. Resolution values are shown in the top left corner and Fourier power spectrums are in each panel's bottom left corner, showing that EMDiffuse generates images with the resolution similar to the GT image and effectively reduces noise without undesirable over-smoothness. The uncertainty value is displayed in the bottom right corner. U, uncertainty value. Scale bar, left, 0.3 μm; right, 0.1 μm.

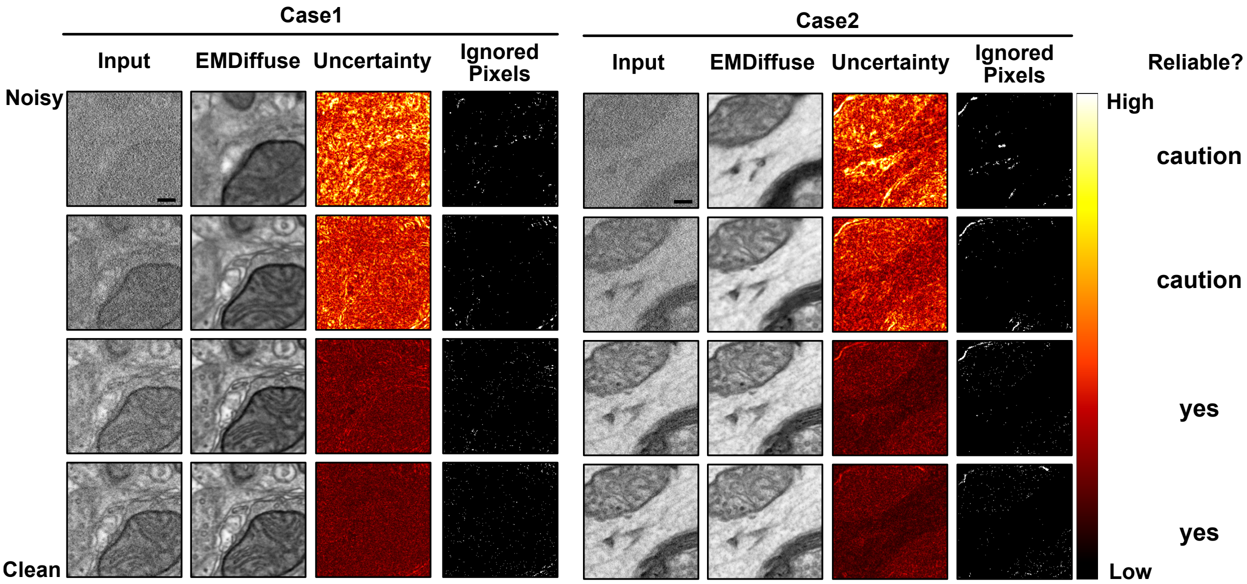

Supplementary Fig. 6 Uncertainty map for self-assessment of prediction reliability. EMDiffuse denoising predictions and uncertainty maps of two example regions with different noise levels. Each row consists of the noise levels, input image, EMDiffuse prediction, uncertainty map and 1% ignored uncertainty pixels consisting of outliers and pixels in the structure boundary. A threshold of 0.12 for the total uncertainty is set for assessing the reliability of the final prediction, as shown in the last column. The caution signifies the potential inaccuracies in the predicted structure, while yes denotes that the prediction is reliable. Scale bar, 0.1 μm.

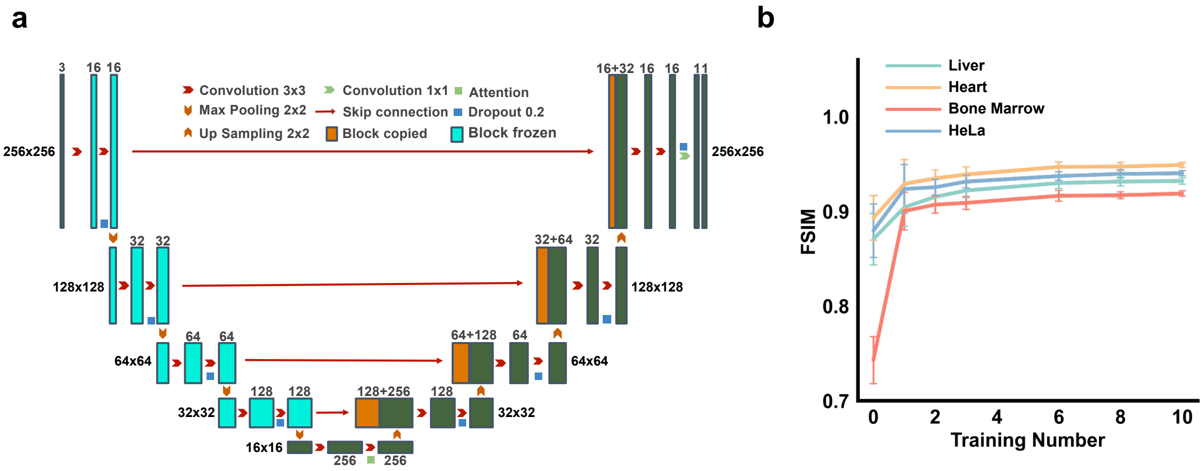

Supplementary Fig. 7 Encoder-frozen transfer learning for EMDiffuse enables fine-tuning with one training pair.

a. Model architecture for EMDiffuse transfer learning. The encoder part of the model is frozen to prevent over-fitting and reduce the requirement for training data. The bottleneck with attention mechanisms is not frozen.

b. FSIM results of EMDiffuse denoising performance using different numbers of transfer learning training images. The proposed half-frozen training approach achieved similar performance using only one training image (3 megapixels) across EM images from three different mouse tissues and one cultured HeLa cell sample, overcoming the limitations of a small fine-tuning dataset (n=10).

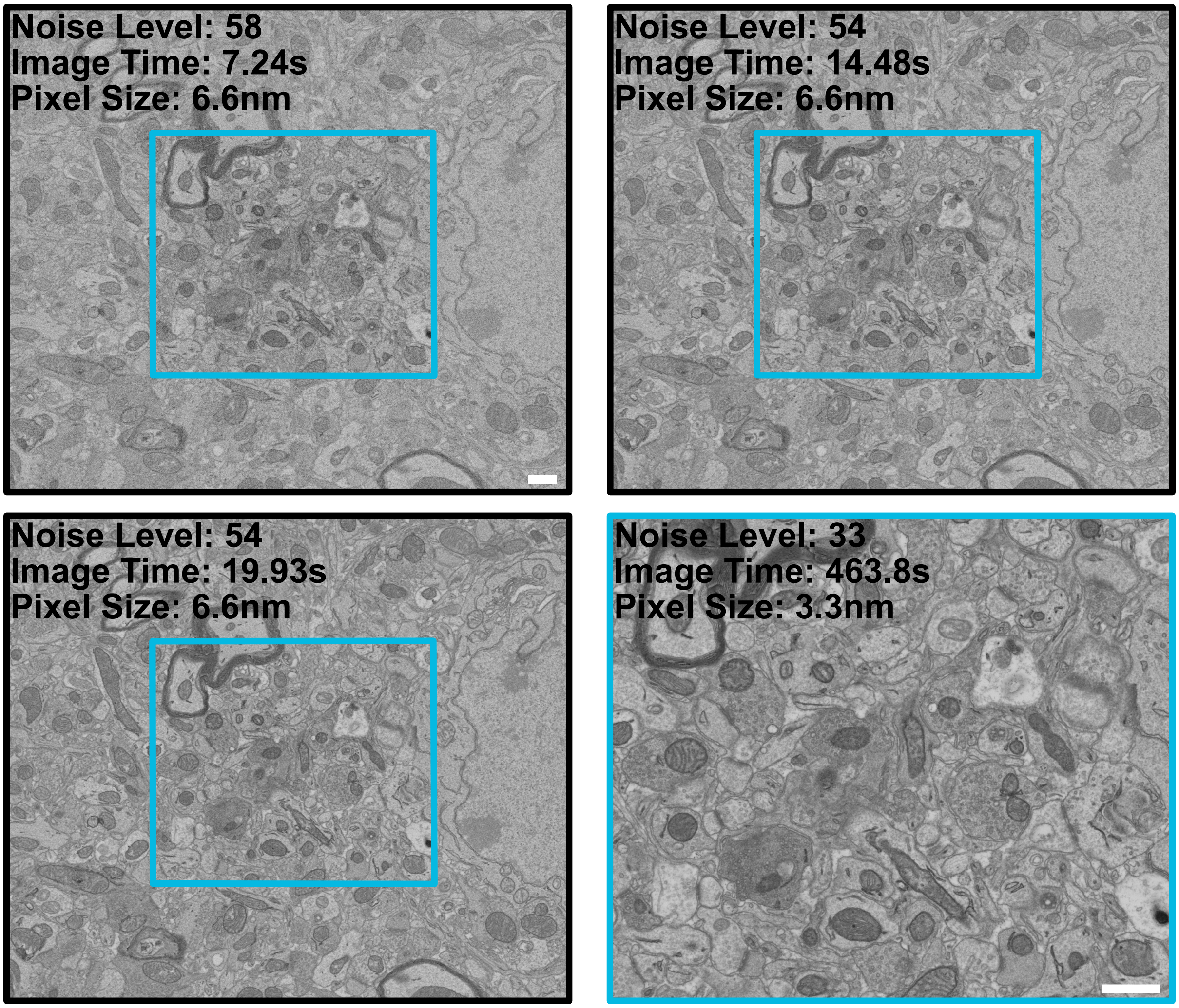

Supplementary Fig. 8 Super-resolution dataset acquisition.
Examples of the source EM images are obtained with a pixel size of 6.6 nm (magnification rate of 20,000x), and the target image is obtained with a pixel size of 3.3 nm (magnification rate of 40,000x) at the central area in the source image. The dataset comprises three dwell times of input images ranging from 2 µs to 6 µs. Scale bar, 0.5 µm.

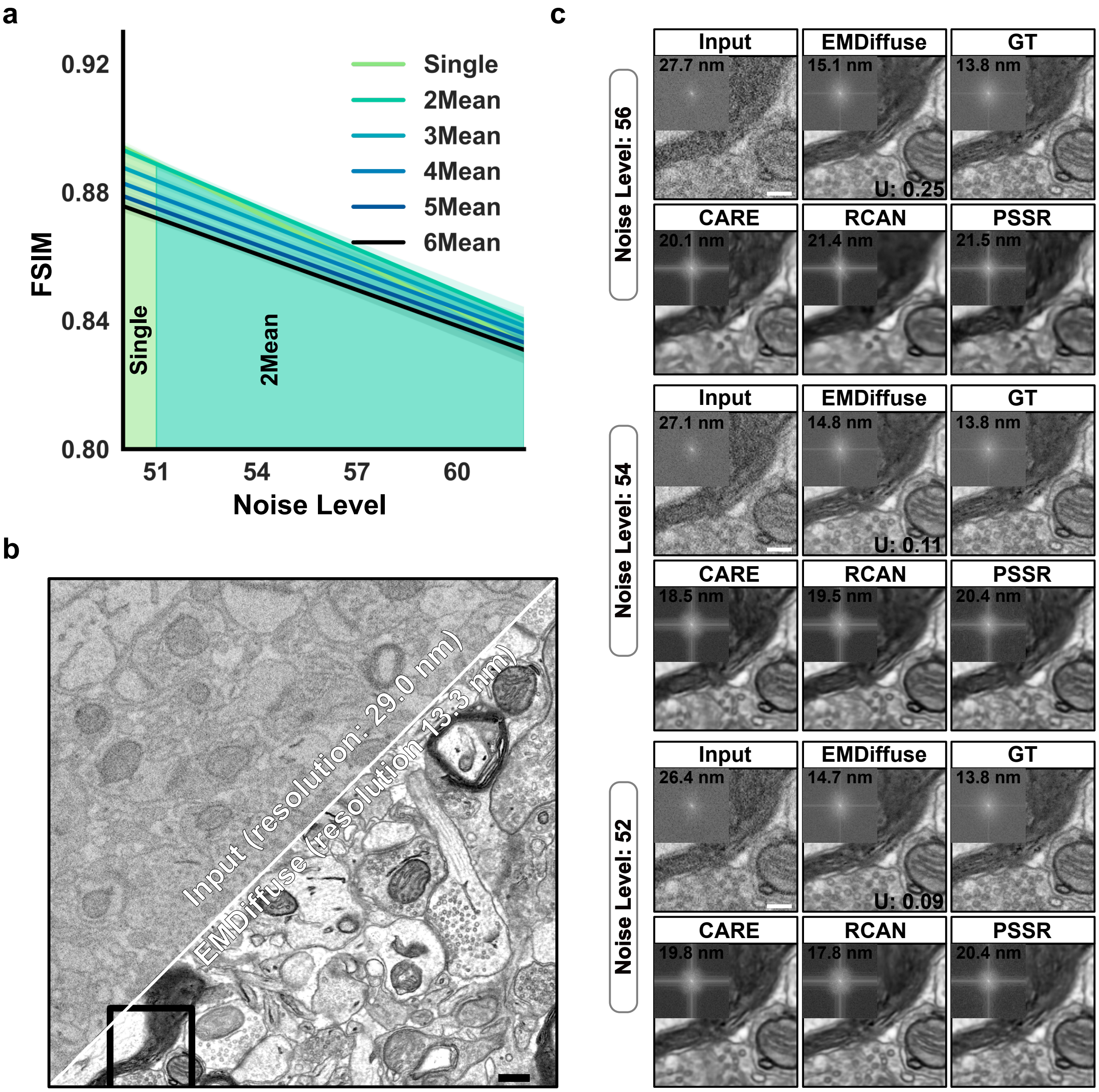

Supplementary Fig. 9 EMDiffuse outperforms other super-resolution methods across various noise levels in structural and perceptual similarities.

a. FSIM metrics of EMDiffuse super-resolution from different noise levels and different mean numbers on the super-resolution dataset. EMDiffuse adaptively selects the appropriate number of images for dynamic mean based on the noise level of the input image to obtain the highest FSIM score.

b. Additional example of EMDiffuse super-resolution result and the corresponding input image.

c. Representative images of one enlarged region of b with GT, different noise levels input and super-resolution predictions from EMDiffuse, CARE, RCAN, and PSSR. Resolution values and Fourier power spectrum are in each panel's top left corner. The uncertainty values are indicated in EMDiffuse prediction results.

U, uncertainty value. Scale bar, (b), 0.3 μm; (c), 0.1 μm.

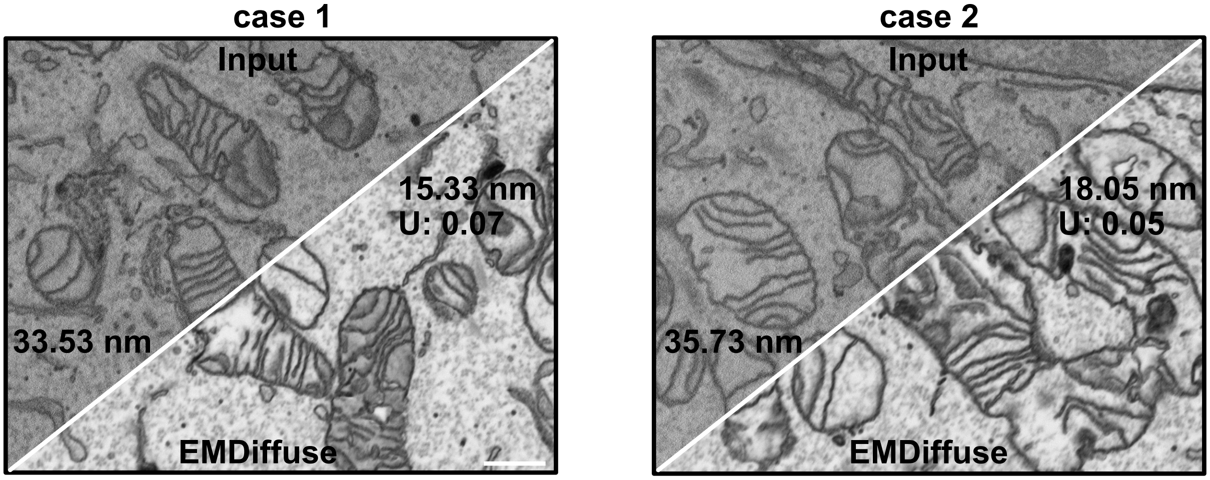

Supplementary Fig. 10 EMDiffuse-r can be easily adapted to cultured HeLa cell images. Examples of EMDiffuse-r transfer learning from mouse brain cortex dataset to cultured HeLa cell sample with a pixel size of 2.1 nm. The model is fine-tuned with one well-aligned pair (3 megapixels) of downsampled noisy and high-quality cultured HeLa cell images. U, uncertainty value. Scale bar 0.15 µm.

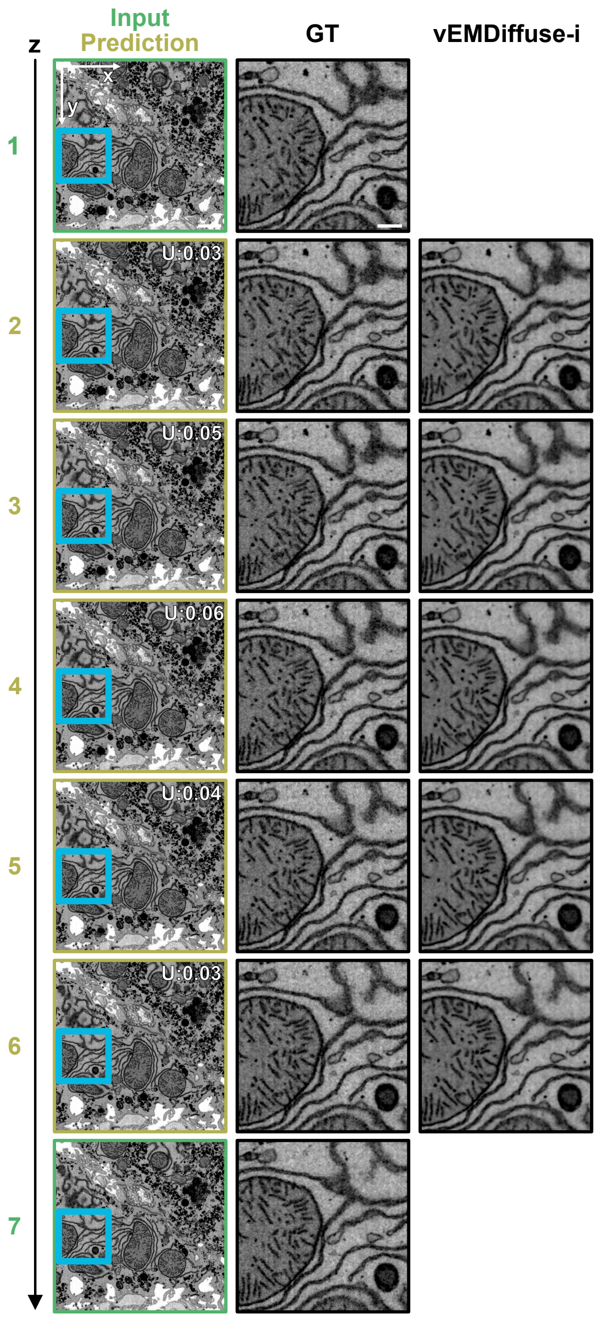

Supplementary Fig. 11 vEMDiffuse-i generates XY Views with isotropic resolution and captures the continuity of organelles along the z-axis in the Openorganelle liver dataset. A series of input and predicted XY views (from 8 nm x 8 nm x 48 nm resolution anisotropic volume) along the z-axis of a liver ultrastructure dataset (jrc_mus-liver). The first column presents the layers of the input (depicted in green) and the vEMDiffuse-i predictions with the uncertainty values (depicted in yellow). The second and third columns showcase layers of the isotropic volume (GT) and vEMDiffuse-i predicted isotropic volume of an enlarged region of the first column. vEMDiffuse-i captures the gradual changes in the ultrastructure of the endoplasmic reticulum (ER), the same as shown in the ground truth slices. U, uncertainty value. Scale bar, first column, 1 μm; second and third columns, 0.3 μm.

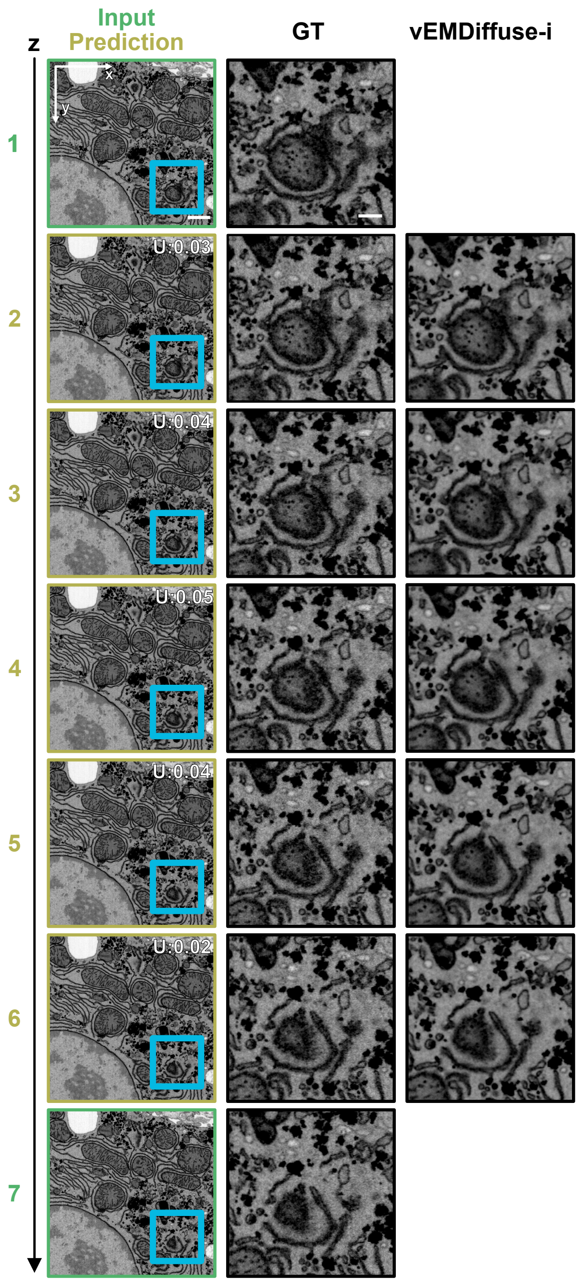

Supplementary Fig. 12 Additional example of the performance of vEMDiffuse-i for generating XY views with isotropic resolution of the Openorganelle liver dataset.

Another series of input and vEMDiffuse-i predicted XY views (from 8 nm x 8 nm x 48 nm resolution anisotropic volume) along the z-axis of the Openorganelle liver dataset (jrc_mus-liver). The first column presents the layers of the input (depicted in green) and the vEMDiffuse-i predictions with the uncertainty values (depicted in yellow). The second and third columns showcase layers of the isotropic volume (GT) and vEMDiffuse-i predicted isotropic volume of an enlarged region of the first column. U, uncertainty value. Scale bar, first column, 1 μm, second and third columns, 0.3 μm.

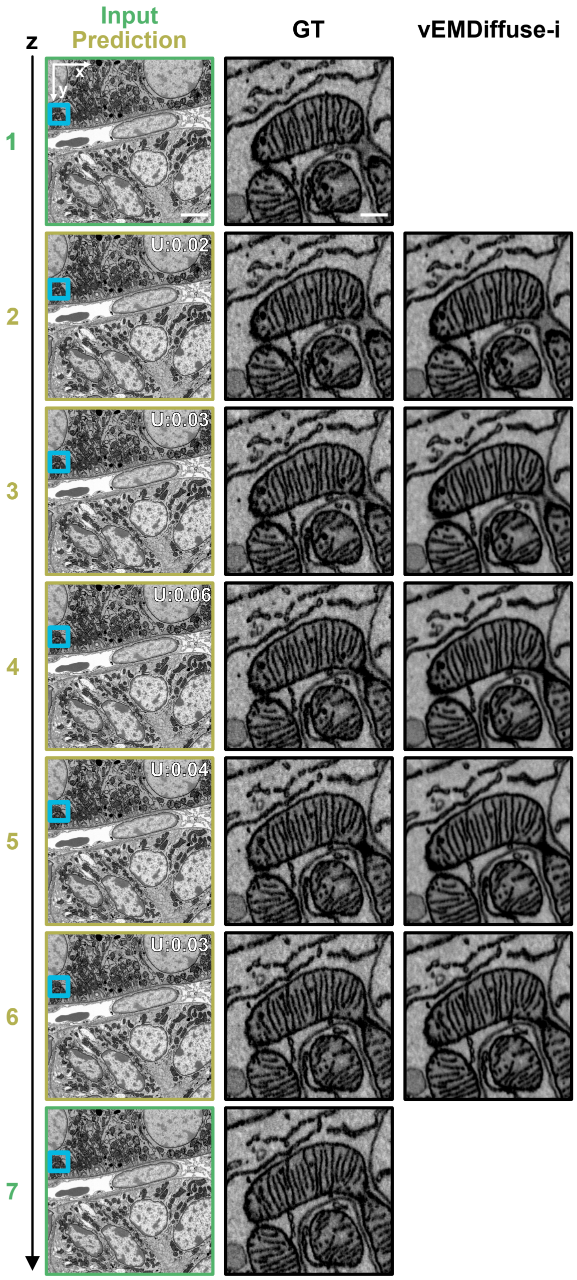

Supplementary Fig. 13 vEMDiffuse-i generates isotropic vEM volumes and captures the continuity of ultrastructure along the z-axis in the Openorganelle kidney dataset.
Shown are a series of input and vEMDiffuse-i predicted XY views (from 8 nm x 8 nm x 48 nm resolution anisotropic volume) with uncertainty values along the z-axis with isotropic resolution of the Openorganelle Kidney Dataset (jrc_mus-kidney). The figure is organized identically to Supplementary Fig. 10b and Supplementary Fig. 11. vEMDiffuse-i's prediction (depicted in yellow) captures the gradual changes of the ultrastructure of the mitochondria and mitochondria cristae as shown in the ground truth. U, uncertainty value. Scale bar, first column, 400 nm; second and third columns, 40 nm.

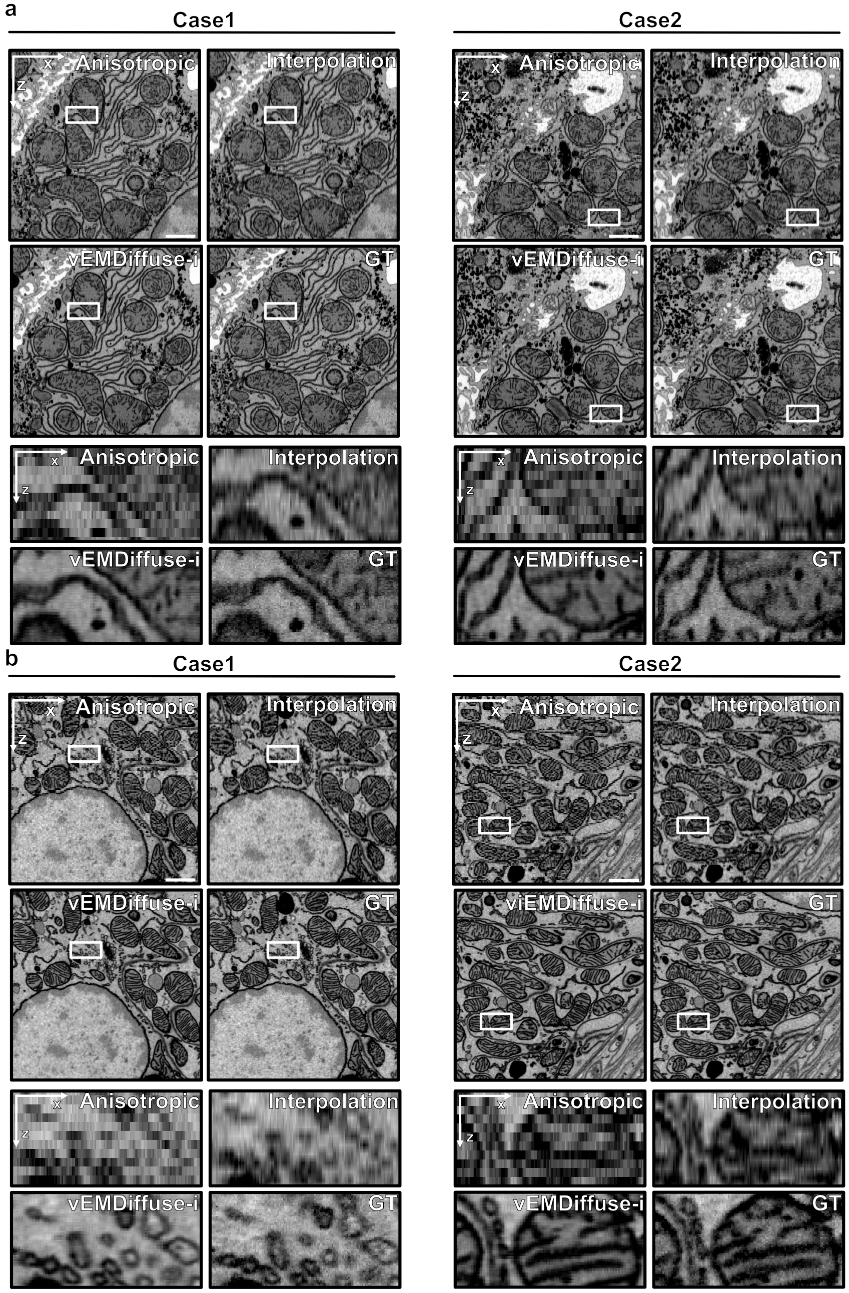

Supplementary Fig. 14 vEMDiffuse-i reconstructs anisotropic volumes into isotropic volumes of two vEM datasets.

a. XZ views of the ultrastructure of a mouse liver. Shown are two XZ view examples and their enlarged regions of the anisotropic volume (8 nm x 8 nm x 48 nm resolution), interpolated volume, vEMDiffuse-i generated volume and isotropic volume (GT, 8 nm x 8 nm x 8 nm resolution) from the Openorganelle liver dataset (jrc_mus-liver).

b. XZ views of the ultrastructure of a mouse kidney. Shown are two XZ view examples and their enlarged regions of the anisotropic volume (8 nm x 8 nm x 48 nm resolution), interpolated volume, vEMDiffuse-i generated volume, and isotropic volume (GT, 8 nm x 8 nm x 8 nm resolution) from the Openorganelle kidney dataset (jrc_mus-kidney).

Scale bar, 1 μm.

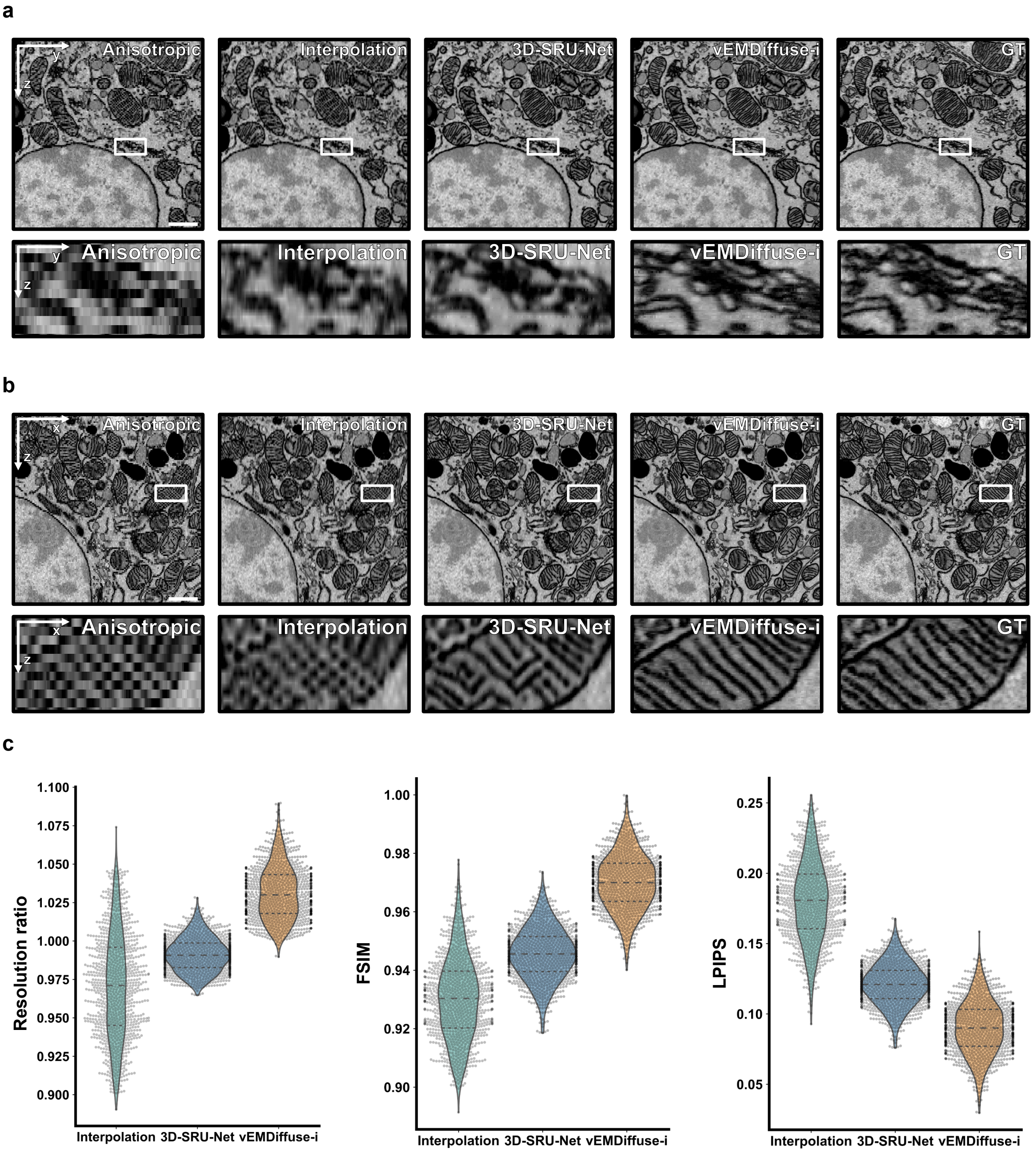

Supplementary Fig. 15 vEMDiffuse-i outperforms other isotropic reconstruction methods.
a, b. Example XZ view and YZ view and enlarged regions of the anisotropic volume (8 nm x 8 nm x 48 nm resolution), interpolated volume (ITK-cubic), 3D-SRU-Net generated volume, vEMDiffuse-i generated volume, and ground truth isotropic volume (GT, 8 nm x 8 nm x 8 nm resolution) on the Openorganelle mouse kidney dataset (jrc_mus-kidney). Scale bar, 1 μm.
c. The violin plots of three metrics (LPIPS, FSIM, and resolution ratio) show the quantitative performance assessment of vEMDiffuse-i for the isotropic reconstruction task compared with ITK-cubic interpolation and 3D-SRU-Net (n=1000). Source data are provided as a Source Data file.

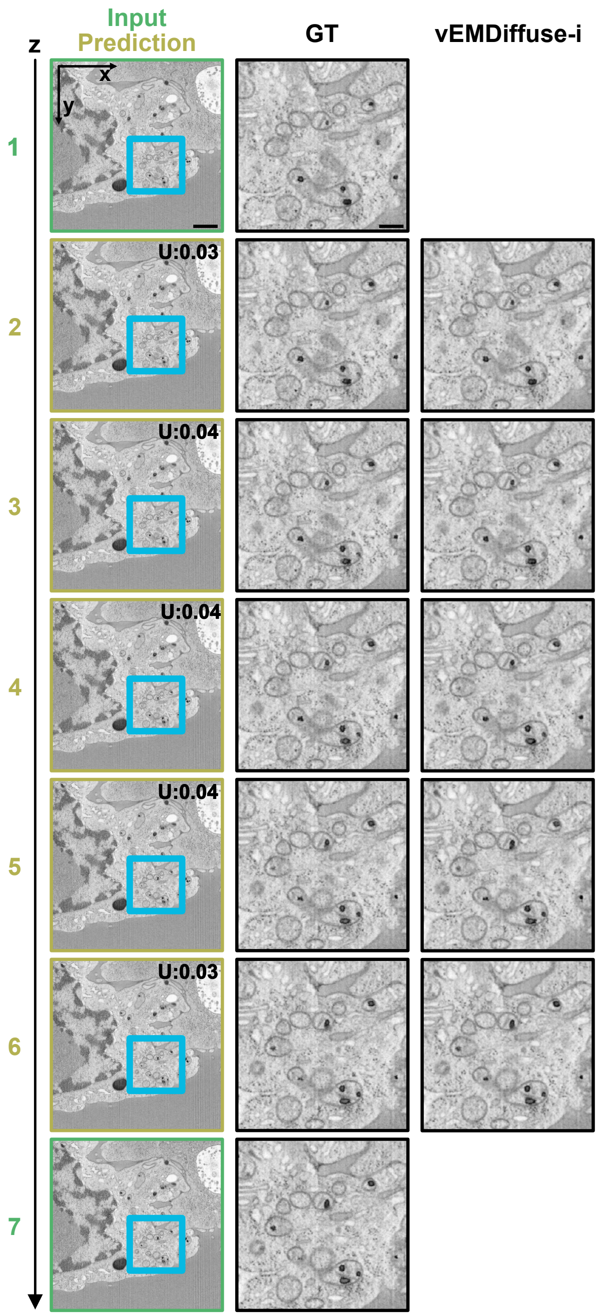

Supplementary Fig. 16 vEMDiffuse-i generates isotropic vEM volumes and captures the continuity of ultrastructure along the z-axis in the T-cell vEM datasets.

Shown are a series of input and vEMDiffuse-i predicted XY views (from 8 nm x 8 nm x 48 nm resolution anisotropic T-cell volume) with uncertainty values along the z-axis with isotropic resolution of the Openorganelle T-cell Dataset (jrc_ctl-id8-2). The figure is organized identically to Supplementary Fig. 10b, Supplementary Fig. 11 and Supplementary Fig. 12. vEMDiffuse-i's prediction (depicted in yellow) captures the gradual changes of the ultrastructure of the organelles as shown in the ground truth. U, uncertainty value. Scale bar, first column, 1 μm; second and third columns, 0.3 μm.

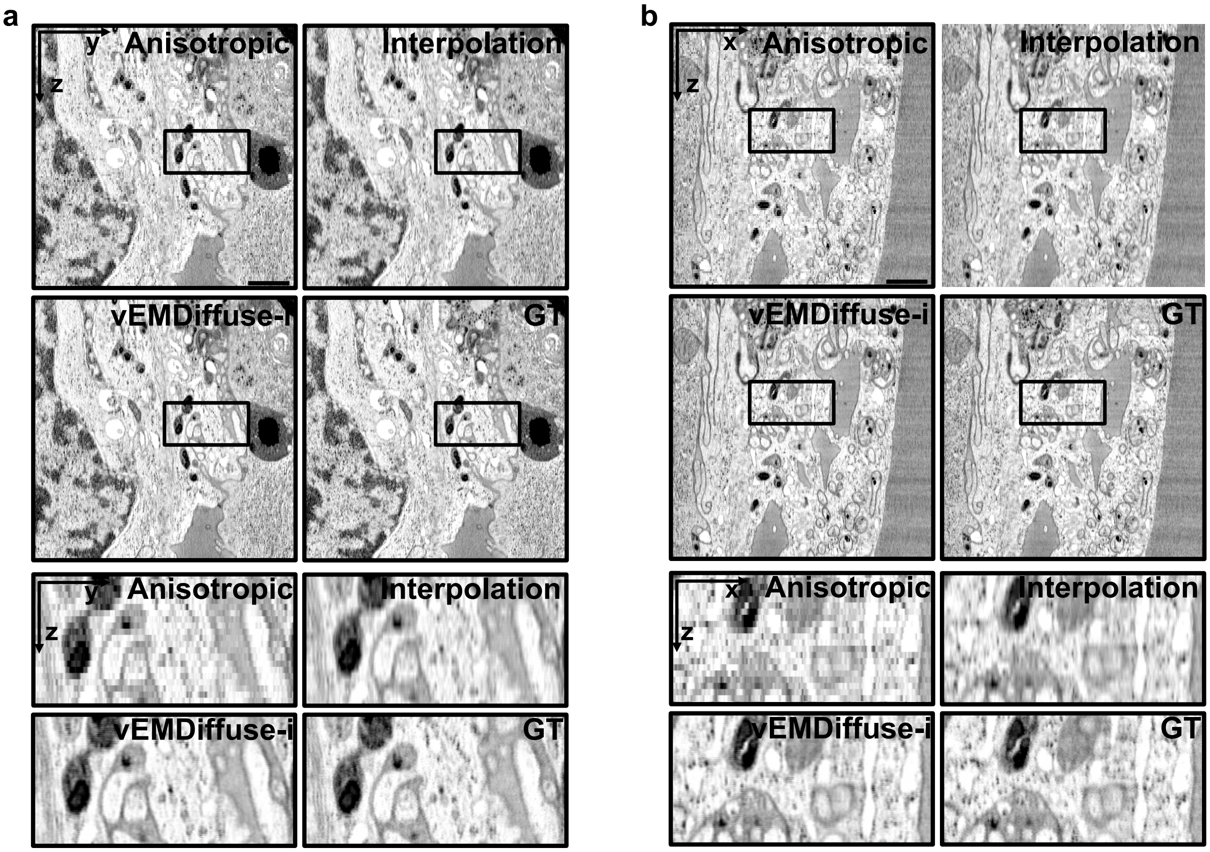

Supplementary Fig. 17 vEMDiffuse-i reconstructs anisotropic volumes into isotropic volumes of T-cell vEM datasets.

a. YZ view of the ultrastructure of a T-cell. Shown are one YZ view example and one enlarged region of the anisotropic volume (8 nm x 8 nm x 48 nm resolution), interpolated volume, vEMDiffuse-i generated volume and isotropic volume (GT, 8 nm x 8 nm x 8 nm resolution) from the Openorganelle T-cell dataset (jrc_ctl-id8-2).

b. XZ view of the ultrastructure of a T-cell. Shown are one XZ view example and their one regions of the anisotropic volume, interpolated volume, vEMDiffuse-i generated volume, and isotropic volume (GT) from the Openorganelle T-cell dataset (jrc_ctl-id8-2).

Scale bar, 1 μm.

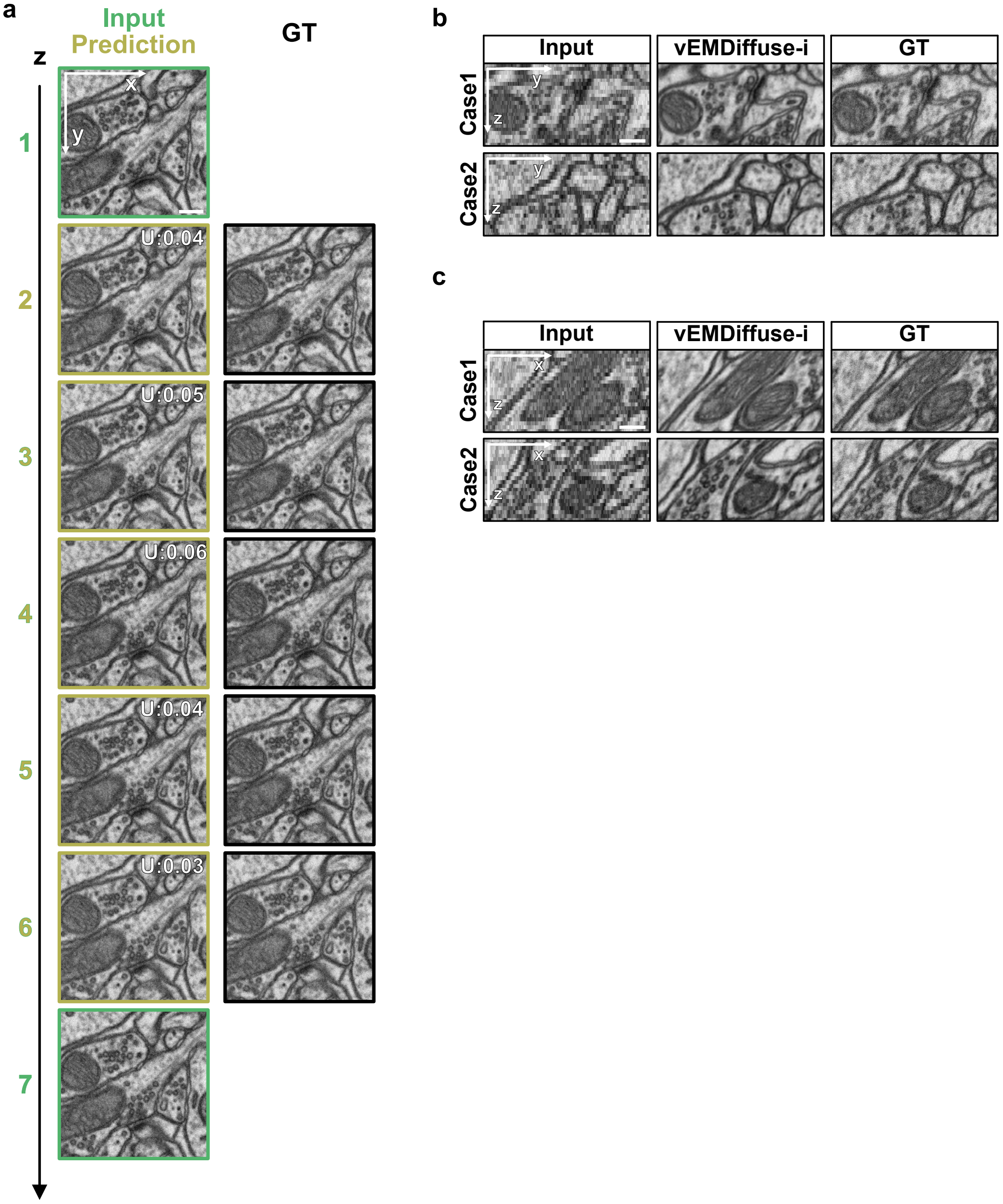

Supplementary Fig. 18 vEMDiffuse-i is transferable for the brain vEM dataset and captures the continuity of neuron ultrastructure.

a. XY view of vEMDiffuse-i generated anisotropic brain volume (5 nm x 5 nm x 30 nm resolution) with uncertainty values and isotropic brain volume from the EPFL brain dataset (5 nm x 5 nm x 5 nm resolution). The first column presents the layers of the input (depicted in green) and the vEMDiffuse-i predictions with the uncertainty values (depicted in yellow). The second column displays the same layers in ground truth isotropic volume. vEMDiffuse-i captures the gradual appearance of vesicles and discriminates proximal membrane structures in neurons along the z-axis.

b and c. YZ view (b) and XZ view (c) of anisotropic brain volume, vEMDiffuse-i generated isotropic volume, and ground truth isotropic volume (GT).

U, uncertainty value. Scale bar, (a), 0.2 μm; (b), 0.1 μm.

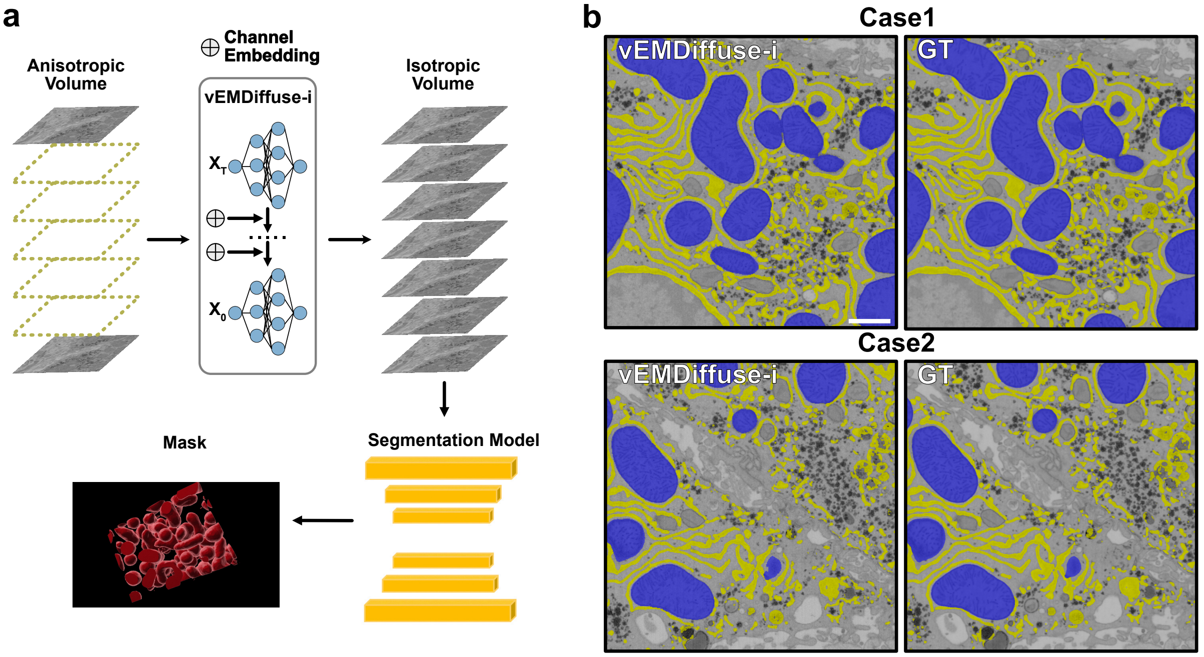

Supplementary Fig. 19 3D reconstruction pipeline and the generation of organelle masks on vEMDiffuse-i reconstructed EM volume and isotropic EM volume.

a. Schematic of the 3D reconstruction pipeline of isotropic vEM datasets generated by vEMDiffuse-i. A segmentation deep learning model is incorporated to segment organelles of interest for the 3D reconstruction of vEM datasets.

b. Examples of segmentation results. Two representative XY view composite images of vEMDiffuse-i generated images and corresponding GT images with the organelle segmentation masks generated with the segmentation network. Blue mask, mitochondria; yellow mask, ER. Scale bar, 1 µm.

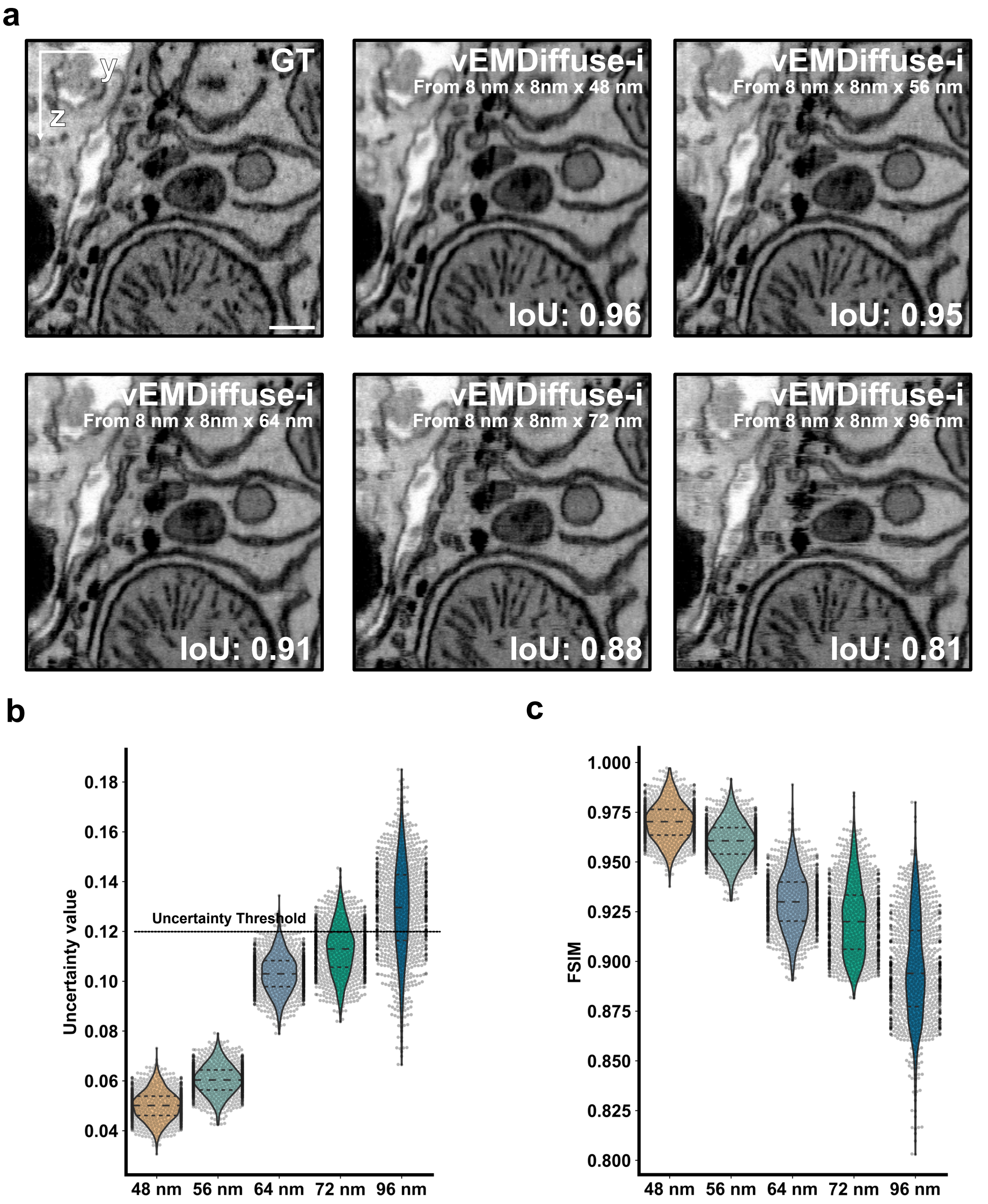

Supplementary Fig. 20 Reliability of vEMDiffuse-i generated volumes from anisotropic volumes with different axial resolutions.

a. Example YZ view of the isotropic volume (8 nm x 8 nm x 8 nm resolution), vEMDiffuse-i generated volume from 8 nm x 8 nm x 48 nm resolution 8 nm x 8 nm x 56 nm resolution, 8 nm x 8 nm x 64 nm, 8 nm x 8 nm x 72 nm, from 8 nm x 8 nm x 96 nm resolution anisotropic volumes downsampled from the Openorganelle mouse liver dataset (jrc_mus-liver). The Intersection over Union (IoU) scores of ER and mitochondria segmentation results are shown in the bottom right corner. Scale bar, 0.3 μm.

b. The violin plots of FSIM metrics of five generated volumes (n=1000).

c. The violin plots of uncertainty values of five generated volumes (n=1000).

Source data are provided as a Source Data file.

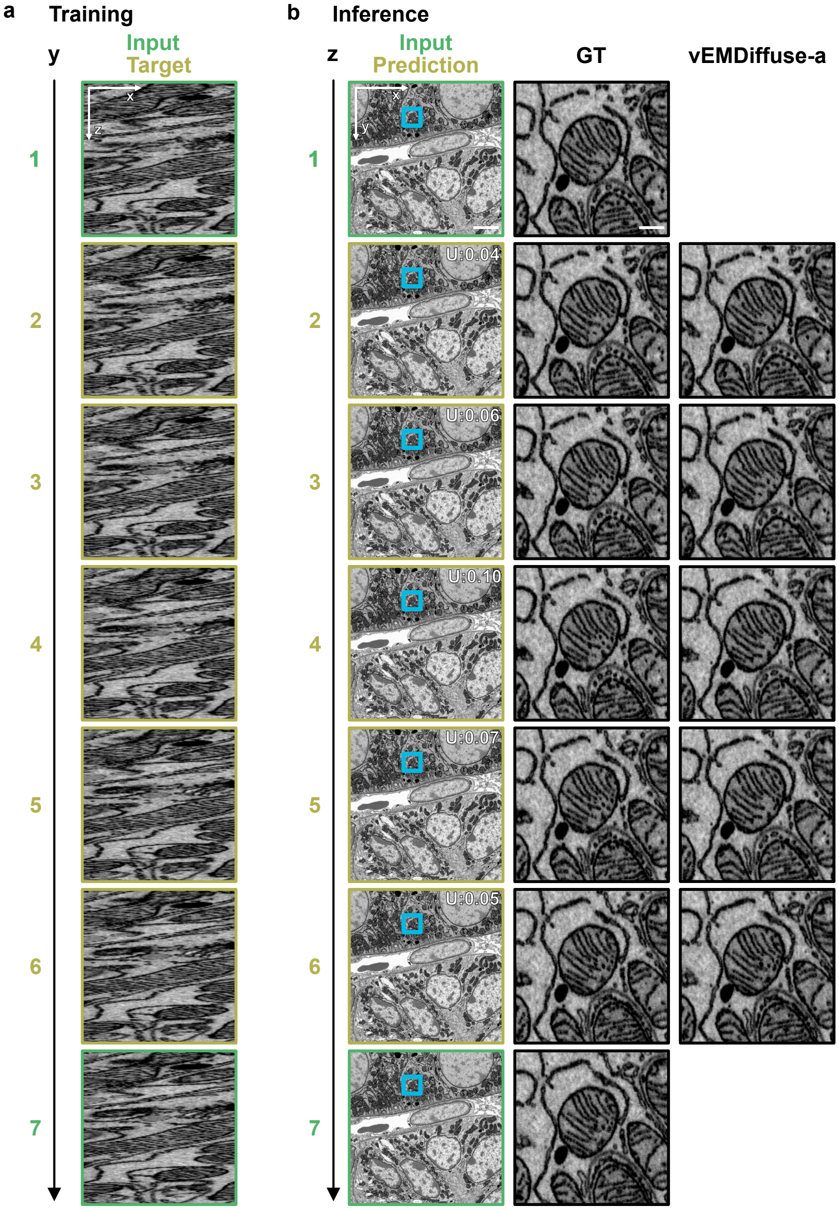

Supplementary Fig. 21 vEMDiffuse-a generates isotropic vEM volumes and captures continuity of ultrastructure along the z-axis with only XZ views as training Data.

a. Example training images. A series of XZ views with uncertainty values along the y-axis of the downsampled Openorganelle kidney dataset (by removing several z slices, 8 nm x 8 nm x 48 nm resolution) is used to train vEMDiffuse-a.

b. Example inference images. A series of XY views along the z-axis generated by vEMDiffuse-a. The first column presents the layers of the input (depicted in *green*) and the vEMDiffuse-a predictions (depicted in *yellow*). The second and third columns showcase layers of the isotropic volume (GT) and vEMDiffuse-a predicted isotropic volume of an enlarged region of the first column. vEMDiffuse-a captures the gradual changes in the ultrastructure of the endoplasmic reticulum (ER) and mitochondria, as shown in the ground truth slices. U, uncertainty value. Scale bar, first column, 400 nm; second and third columns, 40 nm.

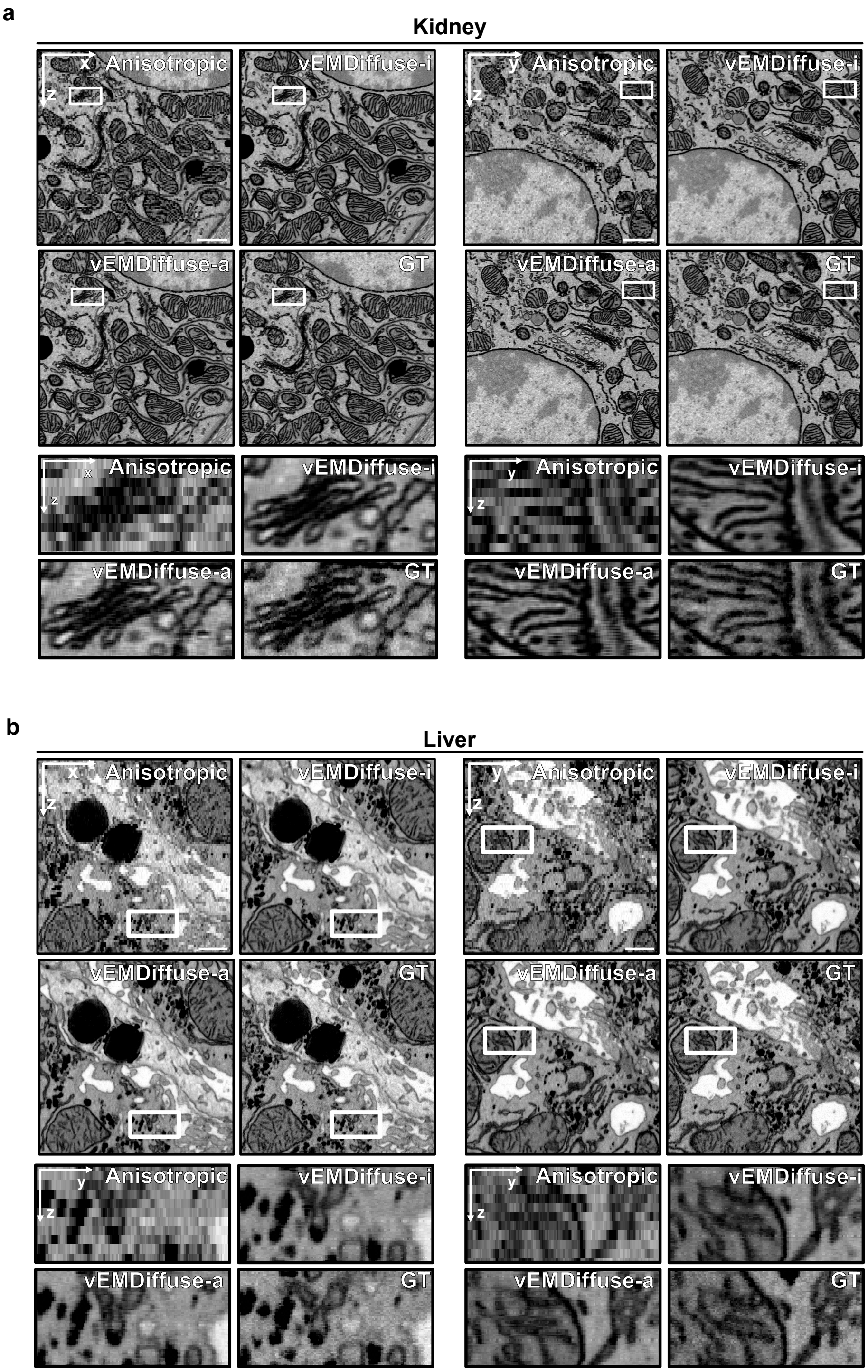

Supplementary Fig. 22 vEMDiffuse-a achieves comparable performance in generating isotropic volumes of vEM datasets with vEMDiffuse-i.

Example XZ view and YZ view and enlarged regions of the anisotropic volume (8 nm x 8 nm x 48 nm resolution), vEMDiffuse-i generated volume, vEMDiffuse-a generated volume, and ground truth isotropic volume (GT, 8 nm x 8 nm x 8 nm resolution) on the Openorganelle kidney dataset (a) and Openorganelle liver dataset (b). Scale bar, (a), 1 μm; (b), 0.6 μm.

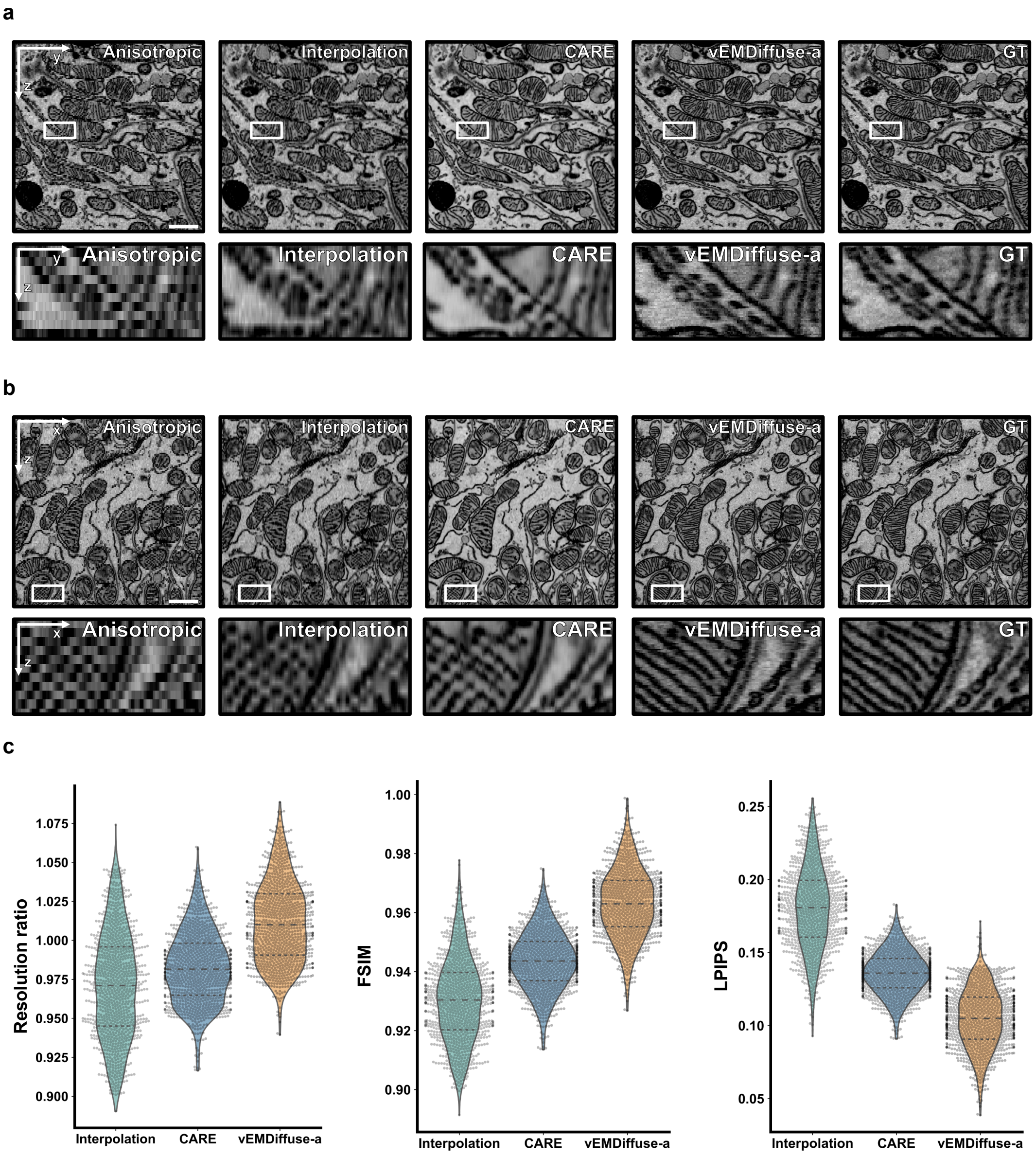

Supplementary Fig. 23 vEMDiffuse-a outperforms other unsupervised isotropic reconstruction methods.

a, b. Example XZ view and YZ view and enlarged regions of the anisotropic volume (8 nm x 8 nm x 48 nm resolution), interpolated volume (ITK-cubic), CARE generated volume, vEMDiffuse-a generated volume, and ground truth isotropic volume (GT, 8 nm x 8 nm x 8 nm resolution) on the Openorganelle mouse kidney dataset (jrc_mus-kidney). Scale bar, 1 μm.

c. The violin plots of three metrics (LPIPS, FSIM, and resolution ratio) show the quantitative performance assessment of vEMDiffuse-a for the isotropic reconstruction task compared with ITK-cubic interpolation and CARE (n=1000). Source data are provided as a Source Data file.

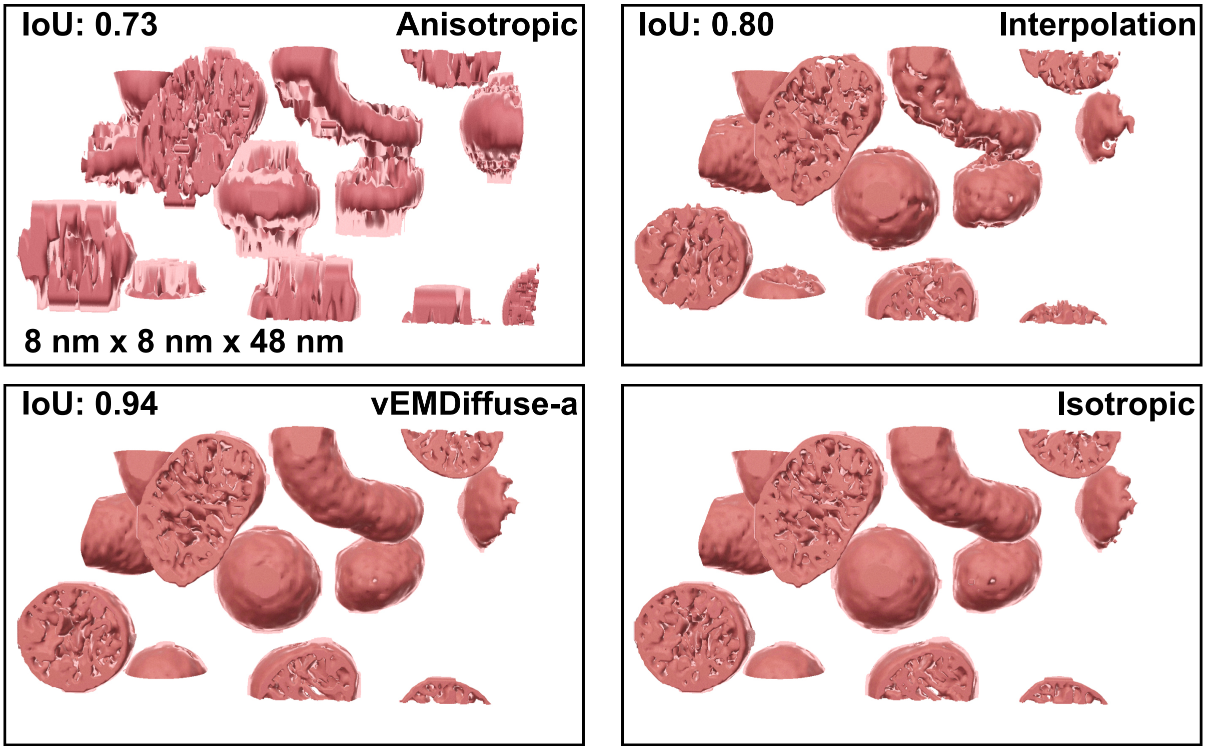

Supplementary Fig. 24 vEMDiffuse-a enhances mitochondria membrane reconstruction of the Openorganelle mouse liver dataset (jrc_mus-liver).
Comparison of the 3D mitochondria membrane reconstruction results between the anisotropic mask (8 nm x 8 nm x 48 nm resolution), segmentation masks of interpolated volume (ITK-cubic), segmentation masks of vEMDiffuse-a generated volume and segmentation masks of the ground truth isotropic volume (8 nm x 8 nm x 8 nm resolution). The Intersection over Union (IoU) score of segmentation masks is calculated against the segmentation mask of the ground truth isotropic volume to avoid the potential influence of the segmentation model.

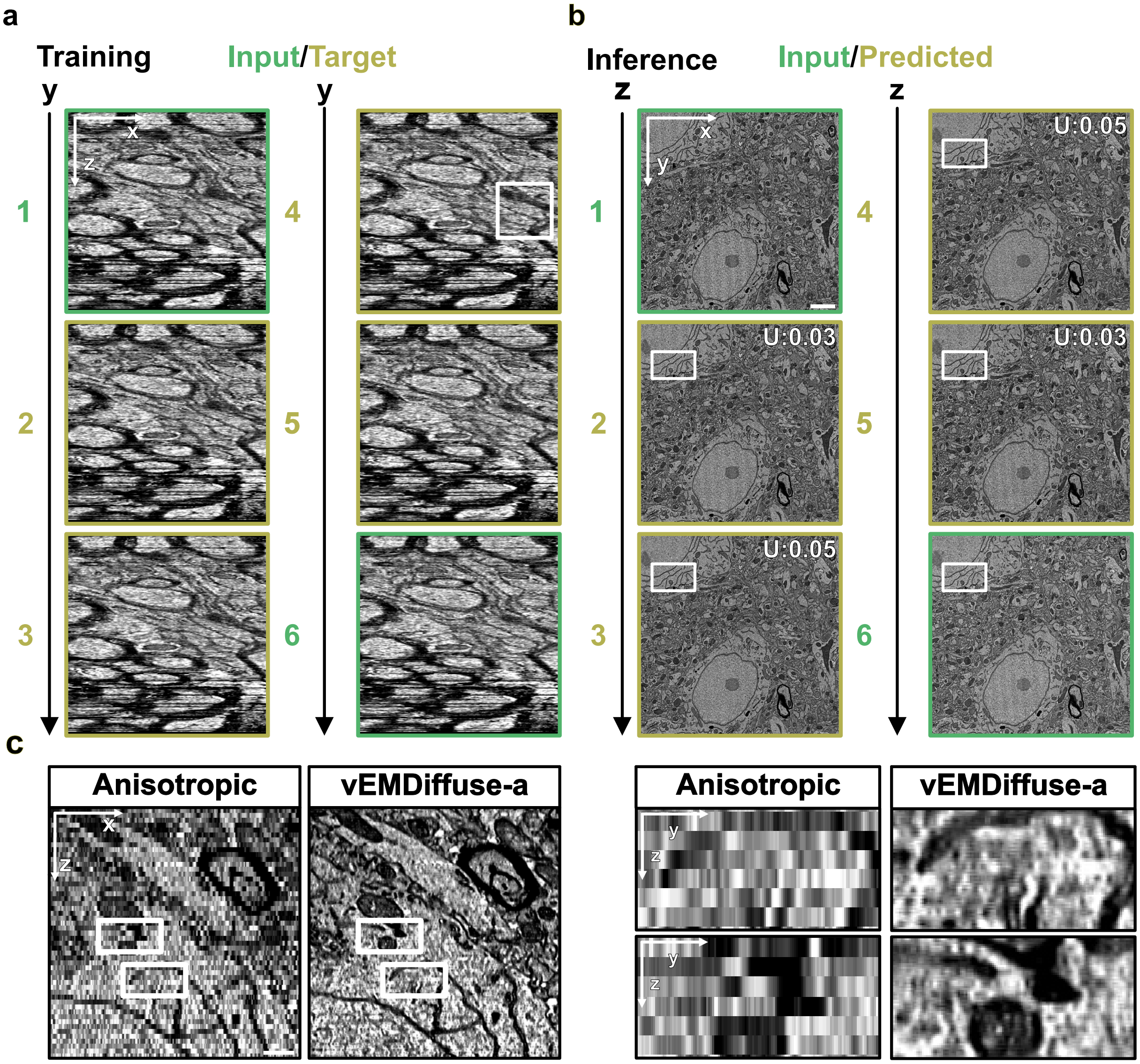

Supplementary Fig. 25 vEMDiffuse-a significantly enhances the axial resolution of the MICrONS multi-area dataset.

a. Example training images examples. A series of XZ views (input images in *green* and target images in *yellow*) along the y-axis of the anisotropic MICrONS multi-area dataset (8 nm x 8 nm x 40 nm resolution) is used to train vEMDiffuse-a.

b. Example inference images. A series of vEMDiffuse-a generated XY views (*yellow*) with uncertainty values and input XY views (*green*) along the z-axis. Scale bar, 1 µm.

c. XZ views and two enlarged regions of the original MICrONS multi-area dataset volume (anisotropic) and vEMDiffuse-a generated volume.

U, uncertainty value. Scale bar, (b), 2 µm; (c), 0.5 µm.

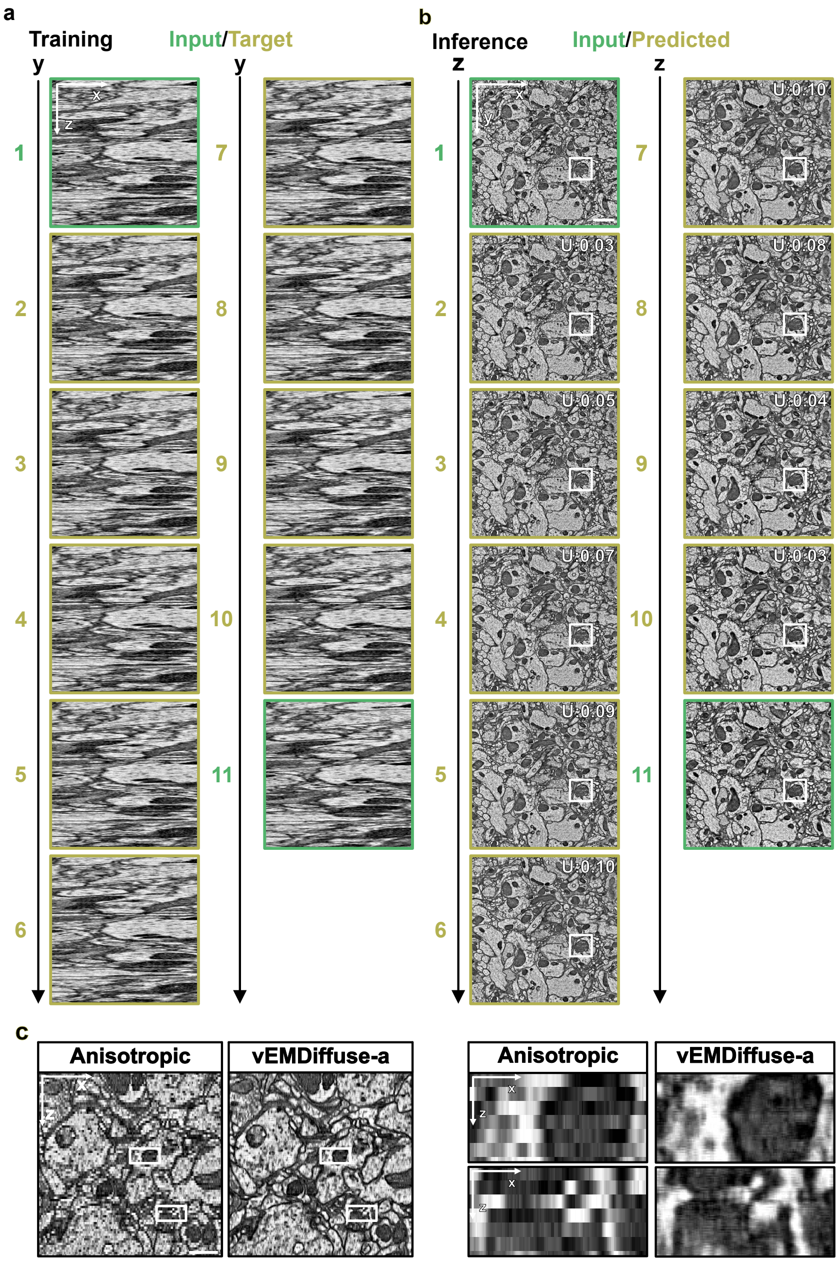

Supplementary Fig. 26 vEMDiffuse-a enhances the axial resolution of the FANC dataset.

a. Example training images examples. A series of XZ views (input images in *green* and target images in *yellow*) along the y-axis of the anisotropic FANC dataset (4 nm x 4 nm x 40 nm resolution) is used to train vEMDiffuse-a.

b. Example inference images. A series of vEMDiffuse-a generated XY views (*yellow*) with uncertainty values and input XY views (*green*) along the z-axis.

c. XZ views and two enlarged regions of the original FANC dataset volume (anisotropic) and vEMDiffuse-a generated volume.

Scale bar, (b), 1.2 μm; (c), 0.5 μm.

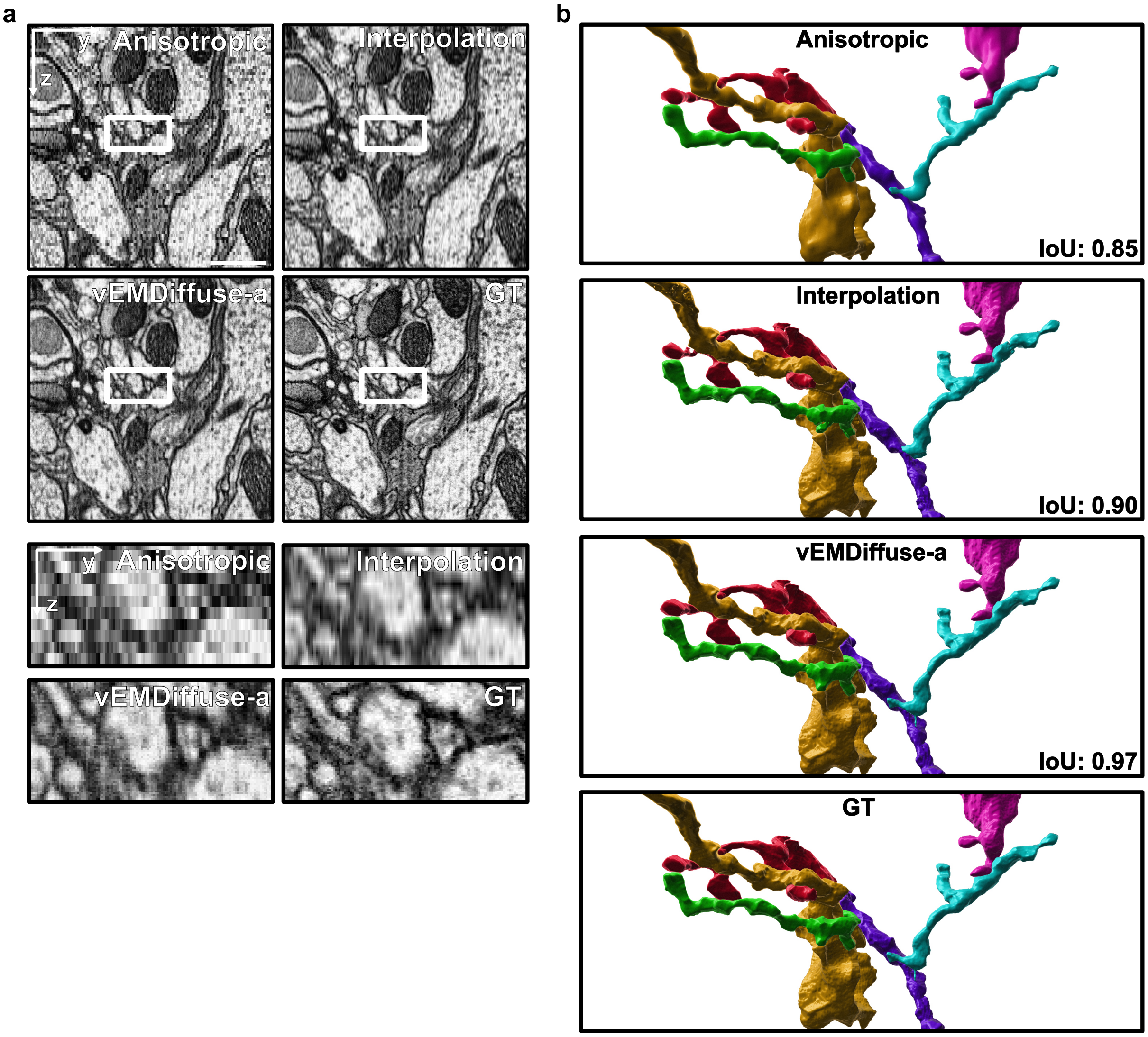

Supplementary Fig. 27 vEMDiffuse-a enhances neurite tracing on the MANC dataset.

a. Example YZ view and enlarged regions of the anisotropic volume (8 nm x 8 nm x 48 nm resolution), interpolated volume, vEMDiffuse-a generated volume, and ground truth isotropic volume (GT, 8 nm x 8 nm x 8 nm resolution) on the MANC dataset. Scale bar, 1 μm.

b. Comparison of the 3D view of neurite tracing results between the anisotropic mask, segmentation masks of interpolated volume, segmentation masks of vEMDiffuse-a generated volume and ground truth mask. The Intersection over Union (IoU) score results are shown in the bottom right corner.

Supplementary Tables

	FSIM	LPIPS	Resolution Ratio
DDPM	0.84	0.14	0.96
DDPM + global attention	0.86	0.11	0.98
DDPM + difficulty aware loss	0.87	0.13	0.97
DDPM + global attention + difficulty aware loss	0.88	0.11	0.99
EMDiffuse (DDPM + global attention + difficulty aware loss + Gaussian blur)	0.89	0.10	1.01

Supplementary Table 1: Ablation study of EMDiffuse-n on mouse brain cortex denoise dataset. Contributions of global attention, difficulty-aware loss, and Gaussian blur data augmentation to EMDiffuse-n's performance were examined. For each study, four experiments were repeated with the mean score reported.

Supplementary Methods

Sample processing and EM imaging

Mice and sample collections. C57BL/6 mice (Male, 10~12-week-old) were provided by Animal Resources Centre (Australia), and the procedures were approved by the Animal Ethics Committee of The University of Western Australia. Following euthanasia, animals were perfused heparin (20U/mL) through the left ventricle, followed by a freshly prepared fixative solution containing 2% paraformaldehyde (PFA) and 2.5% glutaraldehyde in 0.1M sodium cacodylate buffer. Brain and liver were then collected and cut into small pieces (1-3 mm^3), followed by the fixation at 4°C for 3 days. To image the bone marrow, mice tibias were collected and fixed at 4°C for 3 days prior to decalcification in 0.5M ethylenediaminetetraacetic acid (EDTA) for 3 weeks. Small bone fragments were cut for the following preparation.

Cell culture. HeLa cells were maintained in complete DMEM media containing 10% fetal bovine serum (FBS) and 1% penicillin and streptomycin, at 37°C under 5% CO_2. To prepare the cell samples for electron microscopy imaging, cells (5 x 10^4/well) were seeded onto the coverslips in the 24-well-plates and allowed adherence overnight. Next, cells were fixed with 2.5% glutaraldehyde in 0.1M sodium cacodylate buffer (pH 7.4) for 2 hrs and processed for sample preparation.

Sample preparation. Tissues and cells were processed using a modified protocol based on the serial blockface scanning electron microscopy protocol by the National Center for Microscopy and Imaging Research[1]. Briefly, following fixation, tissues and cells were rinsed 5 times for 3 minutes each in 0.1 M sodium cacodylate before 1-hour incubation in 0.1 M sodium cacodylate buffer containing 2% osmium tetroxide and 1.5% potassium ferricyanide at 4°C. Samples were then rinsed and incubated with 1% thiocarbohydrazide for 20 minutes at room temperature. Next, samples were rinsed and incubated with 2% aqueous osmium tetroxide for 30 minutes at room temperature prior to the overnight incubation of 2% uranyl acetate at 4°C. The next day, samples were rinsed and dehydrated through a series of ethanol solutions at increasing concentrations (30%, 50%, 70%, 85%, 95%, 100%) for 10 minutes each, followed by two additional 10-minute incubations in 100% acetone. Tissue samples were then infiltrated with Embed812 resin (Electron Microscopy Sciences) by incubating them in 33% resin (diluted in anhydrous acetone) for 2 hours, 66% resin overnight, and 100% resin overnight. Samples were embedded in fresh resin using polypropylene molds (Electron Microscopy Sciences) and polymerized in an oven for 48 hours at 65°C. After polymerization, samples were removed from the molds, block faces were trimmed, and 500-nm sections were cut using a Leica UC6 ultramicrotome. For cell samples, the coverslips were inverted onto BEEM capsules that were filled with resin, followed by polymerization in an oven for 48 hrs at 60°C. To remove the coverslip, the resin blocks were immersed in liquid nitrogen, and the coverslip can be peeled off using tweezers, and the cells were left on the resin surface. The resin blocks were trimmed, and 500-nm-thickness sections were then cut as described above.

Scanning electron microscopy. Semithin 500-nm-thickness sections were mounted onto silicon wafers. Electron microscopy (EM) images were acquired using the backscattered electron detector using an FEI Verios scanning electron microscope (Thermo Fisher Scientific) with an acceleration voltage was 2 kV. For EMDiffuse-n, the dwell time of the electron dose on each point is changed to adjust the noise level of raw images. In the denoising task, each region is imaged 6 times with

[0.5 μs, 1 μs, 1.5 μs, 2 μs, 4 μs, 36 μs] dwell time with 40,000× magnification with a pixel size of 3.3 nm.

Transfer learning datasets. In transfer learning, we focused on three different tissues: mouse liver, heart, and bone marrow, and cultured HeLa cell sample. The images of three tissue samples have a pixel size of 3.3 nm, and cultured HeLa cell images have a pixel size of 2.1 nm. The dwell time for raw images was set at 2 μs, while for ground truth images, it is 36 μs. These four datasets were imaged using the FEI Verios SEM with an acceleration voltage of 2 kV. The same registration pipeline was applied to transfer learning datasets.

EMDiffuse diffusion process

Denoising Diffusion Probabilistic Models in image quality enhancement.

EMDiffuse's fundamental basis is the Denoising Diffusion Probabilistic Model (DDPM)[2], which belongs to a class of deep generative models. It can generate realistic data samples by reversing a diffusion process. The diffusion process, also known as the forward diffusion process, converts the target data distribution (*e.g.*, the real image distribution) into a tractable probability distribution (*e.g.*, the Gaussian distribution) by gradually adding noise to the data until they become pure noise. DDPM then learns to generate realistic samples from pure random noise by eliminating noise at each step, thereby reversing the diffusion process. In the following section, we will elaborate further on the forward and reverse diffusion process.

Forward process. Given a sample $\mathbf{x}_0$ (e.g., a ground-truth reference image) drawn from the target data distribution $q(\mathbf{x})$, the forward diffusion process, which gradually adds random gaussian noise with T discrete steps, produces a sequence of increasingly noisy samples $\mathbf{x}_1, \mathbf{x}_2, \ldots, \mathbf{x}_T$ sampled from $q(\mathbf{x}_{1:T}|\mathrm{x}_0)$.

$$q(\mathbf{x}_t|\mathbf{x}_{t-1}) = \mathcal{N}\left(\mathbf{x}_t; \sqrt{\alpha_t} \cdot \mathbf{x}_{t-1}, \sqrt{1-\alpha_t} \cdot \mathbf{I}\right) \quad q(\mathbf{x}_{1:T}) = \prod_{t=1}^{T} q(\mathbf{x}_t|\mathbf{x}_{t-1}) \tag{1}$$

where $\mathbf{x}_t$ denotes the noisy data at step t, $\mathbf{I}$ is an identity matrix, and $\alpha_t \in (0,1)$ is the noise schedule parameter that controls the variance of noise at each step t. Specifically, the noise schedule is set so that $\mathbf{x}_T$ becomes pure random noise following a standard normal distribution. For this purpose, the noise schedule parameter α_t initiates from a small value close to zero and grows progressively over time, reaching its maximum value at step T. For this purpose, we adopt the linear schedule as:

$$\alpha_t = 10^{-6} + \frac{t}{T} * (10^{-2} - 10^{-6}) \tag{2}$$

This process is equivalent to sample $\mathbf{x}_t$ at an arbitrary step t from $q(\mathbf{x}_t|\mathbf{x}_0)$:

$$q(\mathbf{x}_t|\mathbf{x}_0) = \mathcal{N}(\mathbf{x}_\mathrm{t}; \sqrt{\bar{\alpha}_t}\mathbf{x}_0\mathbf{x}_\mathbf{0}, (1-\bar{\alpha}_t)\mathbf{I}; \quad \text{where} \quad \bar{\alpha}_t = \prod_{i=1}^{t} \alpha_i \tag{3}$$

Reverse diffusion process. The reverse diffusion process uses a parametric model, i.e., a neural network, with parameters θ to invert the diffusion process and generates high-quality samples $\mathbf{x}_0$ from pure gaussian random noise $\mathbf{x}_T$. In our setting, we include the raw input image $\mathbf{c}_r$ (e.g., noisy input image) as a condition for the reverse diffusion process, which becomes a conditional process that can be expressed as below,

$$p_\theta(\mathbf{x}_{0:T}|\mathbf{c}_r) = p(\mathbf{x}_T)\prod_{t=1}^{T} p_\theta(\mathbf{x}_{t-1}|\mathbf{x}_t, \mathbf{c}_r) \tag{4}$$

$$p_\theta(\mathbf{x}_{t-1}|\mathbf{x}_t, \mathbf{c}_r) = \mathcal{N}\left(\mathbf{x}_{t-1}; \boldsymbol{\mu}_\theta(\mathbf{x}_t, t, \mathbf{c}_r), \boldsymbol{\Sigma}_\theta(\mathbf{x}_t, t, \mathbf{c}_r)\right) \tag{5}$$

where $\boldsymbol{\mu}_\theta$ and $\boldsymbol{\Sigma}_\theta$ are denoising functions realized by a deep neural network with parameters θ to predict the mean and covariance matrix and t is the reverse step index.

The reverse process is trained to maximize a variational lower bound, e.g., $-L_{VLB}(\mathbf{x}_0)$ of the log data likehood, i.e., $\log(p_\theta(\mathbf{x}_0))$, which essentially targets at matching the joint distribution of the forward process, i.e., $q(\mathbf{x}_{1:T}|\mathbf{x}_0)$, as:

$$\mathrm{E}_{q(\mathbf{x}_0)} \log p_\theta(\mathbf{x}_0|\mathbf{c}_r) \geq \mathrm{E}_{q(\mathbf{x}_{0:T})} \log \frac{q(\mathbf{x}_{1:T}|\mathbf{x}_0)}{p_\theta(\mathbf{x}_{0:T}|\mathbf{c}_r)} = -L_{VLB}(\mathbf{x}_0|\mathbf{c}_r), \tag{6}$$

$$p_\theta(\mathbf{x}_0|\mathbf{c}_r) = \int p_\theta(\mathbf{x}_{0:T}|\mathbf{c}_r) d\mathbf{x}_{1:T} \tag{7}$$

By minimizing $L_{VLB}(\mathbf{x}_0|\mathbf{c}_r)$, the network parameters θ are optimized.

Following DDPM[2], we use a non-learnable fixed covariance matrix and adopt the reverse process parameterization of $\boldsymbol{\mu}_\theta$ as:

$$\boldsymbol{\mu}_\theta(\mathbf{x}_t, t, \mathbf{c}_r) = \frac{1}{\sqrt{\alpha_t}}\left(\mathbf{x}_t - \frac{1-\alpha_t}{\sqrt{1-\overline{\alpha}_t}} \boldsymbol{\epsilon}_\theta(\mathbf{x}_t, t, \mathbf{c}_r)\right) \tag{8}$$

which uses a network $\boldsymbol{\epsilon}_\theta$ to predict the noise. DDPM empirically found that a simplification of $L_{VLB}(\mathbf{x}_0|\mathbf{c}_r)$ yield better sample quality in practice. We thus adopt the simplified training objective:

$$\begin{aligned} L_{\text{simple}} &= \mathrm{E}_{\mathbf{x}_0, t\sim[1,T], \boldsymbol{\epsilon}} ||\boldsymbol{\epsilon} - \boldsymbol{\epsilon}_\theta(\mathbf{x}_t, t, \mathbf{c}_r)||^2 \\ &= \mathrm{E}_{\mathbf{x}_0, t\sim[1,T], \boldsymbol{\epsilon}} ||\boldsymbol{\epsilon} - \boldsymbol{\epsilon}_\theta(\sqrt{\overline{\alpha}_t}\mathbf{x}_0 + \sqrt{1-\overline{\alpha}_t}\boldsymbol{\epsilon}, t, \mathbf{c}_r)||^2 \end{aligned} \tag{9}$$

Where $\boldsymbol{\epsilon} \sim \mathcal{N}(0, 1)$.

UDiM network architecture

U-Net architecture. In our approach, we employed a U-Net[3] architecture for $\boldsymbol{\epsilon}_\theta(\mathbf{x}_t, t, \mathbf{c}_r)$ akin to the one implemented in DDPM (Supplementary Fig. 2). U-Net is particularly well-suited for image denoising and super-resolution tasks, owing to its ability to efficiently capture both local and global contextual information from input images. The U-Net architecture consists of two main components: the encoder (Contracting Path) and the decoder (Expanding Path). These two elements are interconnected through skip connections, which facilitate the propagation of high-resolution spatial information from the encoder to the decoder. This feature enables U-Net to

generate fine-grained details in the resulting image, which is crucial for tasks such as image denoising and super-resolution.

Global attention. Drawing inspiration from SR3[4], we incorporated a global attention layer, akin to the self-attention mechanism in transformers, into the U-Net architecture. This enhancement boosted the model's performance by allowing it to capture long-range dependencies and contextual information across the entire input image. Specifically, the global attention layer is placed at the bottleneck of the U-Net i.e., between the encoder (contracting path) and the decoder (expanding path), to reduce the computation costs.

A global attention layer operates as follows. Firstly, we use three linear layers to transform the input feature map, which is derived from the contracting path. This transformation produced the Query ($Q \in R^{H\times W\times C}$), Key ($K \in R^{H\times W\times C}$) and Value ($V \in R^{H\times W\times C}$) embeddings, where H and W represent the spatial height and width of the feature map, and C represents the feature dimension. Next, we compute the attention as:

$$\text{Attention}(Q,K,V) = \text{softmax}\left(\frac{QK^T}{\sqrt{C}}\right)V \tag{10}$$

where the "softmax" operation normalizes the attention weights, resulting in a summation of 1. The attention module refines the feature of a single pixel location by softly aggregating the information from all spatial locations based on their similarities. This process enables the features to effectively capture global information (Supplementary Table 1).

Denoising baseline implementation details

CARE[5] software was installed from GitHub (https://github.com/CSBDeep/CSBDeep). The configurations were consistent with the prior application of CARE to SEM[6]. The depth of the network was 2. The batch size was 16. The initial learning rate was 4e-4. The mean absolute error (MAE) was used as the loss function.

PSSR[7] algorithm was sourced from GitHub (https://github.com/BPHO-Salk/PSSR). Given our pre-acquisition of noisy images and our registration pipeline, the crapper intended for generating noisy EM images for training within PSSR was skipped. The training hyperparameters followed the PSSR manuscript and GitHub guidelines. The mean-squared error loss (MSE) was used as the loss function. The batch size was 64. The One Cycle Policy[8] was adopted as the learning rate schedule with the maximum learning rate set as 4e-4 and stochastic gradient descent with restarts (SGDR) as the optimizer[9].

RCAN[10] was installed from the BasicSR package (https://github.com/XpixelGroup/BasicSR), a compendium of popular super-resolution algorithms. The upsample scale of the model was set as 1. The Adam optimizer[11] with an initial learning rate of 1e-4 was employed to train RCAN. The batch size was 64. The MSE was used as the loss function. The learning rate was halved whenever the validation loss plateaued for over 10 epochs.

Noise2Noise[12] was adopted from GitHub (https://github.com/joeylitalien/noise2noise-pytorch). Notice that our dataset contained images with varying noise within identical regions. This allowed us to use paired noisy images instead of manually adding different noise to images. The batch size

was 64. The Adam optimizer with an initial learning rate of 1e-4 was employed to train the model. The batch size was 64. The MSE was used as the loss function. The learning rate schedule was the same as that of RCAN.

Noise2Void[13] was modified based on GitHub (https://github.com/juglab/n2v). The batch size was 64. The Adam optimizer with an initial learning rate of 4e-4 was employed to train the model. The MSE was employed as the loss function. The learning rate schedule was the same as that of RCAN.

Evaluation metrics

Noise level measurement. In the data acquisition process, the dwell time of the electron dose was adjusted to alter the noise level. However, using dwell time directly to represent noise level of raw images was not universally applicable across different microscopes and tissues. To quantitatively measure the noise level of each raw image, we employed a Blind/Reference-free Image Spatial Quality Evaluator (BRISQUE)[14]. This metric is designed to quantify an image's perceptual quality of an image by examining the naturalness or statistical regularities present in the image. It can effectively evaluate the quality of images subjected to distortions such as compression, noise, or blurring, without requiring an original, undistorted reference image. We used this metric to evaluate the noise level of raw images during inference to optimize the prediction generation method.

PSNR. Peak Signal-to-Noise Ratio (PSNR) is a metric for measuring the quality of generated images with respect to their original ground-truth reference images[5, 15]. It is calculated by comparing the peak signal power (e.g., maximum image intensity) and noise power. PSNR is mostly measured by the mean square error.

$$PSNR = 10 * \log_{10}(\frac{MAX_R^2}{MSE}) \ , \quad MSE = \frac{1}{mn}\sum_{i=1}^{m}\sum_{j=1}^{n}[R(i,j) - I(i,j)]^2 \qquad (11)$$

Where I refers to the generated image and R refers to the ground truth image. m and n are the width and height of the image. MAX_R denotes the maximum pixel value of the ground-truth reference image R. PSNR quantifies the pixel-wise intensity mean square error between the generated image and the ground truth image. A higher PSNR means better image quality. However, it is susceptible to being influenced by noise in the reference image. In electron microscope imaging, it is not feasible to capture noise-free images as ground truth (Figure 1b, Figure 2b). Thus, the pixel-wise mean square error and PSNR scores (error maps in Supplementary Fig. 4) cannot necessarily account for high-quality results due to the impact of random noise. While baseline models, due to the L1 or L2 training loss, predict the average/median of all possible target data distributions for approximating minimum mean square error (MMSE) of solution distribution, which induces smoothness in the prediction and alleviates the influence of noise in reference ground-truth when calculating PSNR. Therefore, we use the Feature Similarity Index Measure (FSIM)[16] and Learned Perceptual Image Patch Similarity (LPIPS)[17] which emphasize structural quality and align well with human judgment. Details of FSIM and LPIPS are discussed as follows.

FSIM. FSIM[16] is a perceptual quality metric inspired by the human visual system. It assesses the similarity between images by examining their low-level features, such as phase congruency and gradient magnitude. It is calculated as follows:

$$FSIM(\mathrm{x},\mathrm{y}) = \frac{\sum_{\{i=1\}}^{N}\sum_{\{j=1\}}^{M} w_{\{i,j\}}(x,y) * S_{(i,j)}(x,y)}{\sum_{\{i=1\}}^{N}\sum_{\{j=1\}}^{M} w_{\{i,j\}}(x,y)} \quad (12)$$

$$S_{i,j}(x,y) = \frac{2PC(x) * PC(y) + T}{PC^{2(x)} * PC^{2(y)} + T} * \frac{2G(x) * G(y) + T}{G^{2(x)} * G^{2(y)} + T} \quad (13)$$

$$w_{\{i,j\}}(x,y) = \max\left(PC(x), PC(y)\right) \quad (14)$$

Where N and M are the height and width of the image respectively. $S(i,j)(x,y)$ denotes the similarity measure between the phase congruency and gradient magnitude features of the pixels (i, j) in images x and y. $PC(x)$ represents phase congruency calculated with the filter proposed ealier[18]. Phase congruency employs the phase of image Fourier transform to capture edges and other prominent structures in an image without being influenced by changes in lighting or contrast. $G(x)$ is the gradient magnitude calculated by the Sobel operator[19]. Gradient magnitude, on the other hand, reflects local variations in intensity or color within an image, offering insight into edges, textures, and intricate details.$w_{\{i,j\}}(x,y)$ denotes the perceptible significance of each pixel which is decided by the maximum value of phase congruency between two images. FSIM is specifically designed to capture the structural and textural information in images, which is a vital aspect of practical EM applications in biology. By concentrating on these features, FSIM delivers a more precise metric of structural similarity between images than PSNR[20, 21].

LPIPS. LPIPS[17] is a perceptual similarity metric that leverages deep neural networks. It is trained on an extensive dataset annotated by humans, which allows it to capture perceptual differences more precisely between images as perceived by human observers. LPIPS prioritizes the similarity of deep image features over pixel-wise differences, resulting in improved robustness and alignment with human perception. Consequently, LPIPS has become a widely adopted quality measurement metric in cutting-edge computer vision researches[22, 23].

Resolution Ratio. We compute the resolution of input, generated image, and GT using image decorrelation analysis[24]. The image decorrelation analysis cross-correlates the normalized Fourier transform with the original image in Fourier space using Pearson correlation. By repeating this calculation while applying a binary circular mask of radius $r\epsilon[0,1]$ on normalized Fourier transform, the decorrelation function is computed.

$$d(r) = \frac{\int\int Re\{I(\boldsymbol{k})I^{*}{}_{n}(\boldsymbol{k})M(k,r)\}dk_x dk_y}{\sqrt{\int\int|I(\boldsymbol{k})|^2\, dk_x dk_y \int\int|I_n(\boldsymbol{k})M(\boldsymbol{k},r)|^2\, dk_x dk_y}} \quad (15)$$

where $I(k)$ denotes the Fourier transform of the input image, $I_n(k)$, normalized $I(k)$, k = [kx, ky], Fourier space coordinates I(k), and M(k;r), the binary mask on frequency domain of radius r. The input image undergoes total N_g high-pass filters, ranging from mild to intense, to reduce low-frequency energy. For each processed image, it calculates a decorrelation function and extracts the peak position labeled as r_i (local maximum).

$$Resolution = \frac{2 * \,pixel\ size}{\max\left(\mathrm{r}_0, \dots, \mathrm{r}_{\mathrm{N_g}}\right)} \quad (16)$$

Then we computed the resolution ratio:

$$Resolution\ Ratio = \frac{GT\ Resolution}{Output\ Resolution} \tag{17}$$

Fourier Ring correlation. The Fourier ring correlation between predictions and ground truth images is plotted using the FIJI plugin (https://imagej.net/plugins/fourier-ring-correlation). The Fourier ring correlation plot reflects the correlation between two images in different spatial frequency components.

$$FRC(r) = \frac{\sum_{r_i \in r} F_1(r_i) * F_2(r_i)^*}{\sqrt{\sum_{r_i \in r} |F_1(r_i)|^2 \ * \sum_{r_i \in r} |F_2(r_i)|^2}} \tag{18}$$

where F_1 and F_2 are the Fourier transform of generated image and ground truth image. $F_1(r_i)$ refers to pixels on the perimeter of circles of constant spatial frequency with magnitude r.

Isotropic reconstruction baseline implementation details

3D-SRU-Net training: To our knowledge, no official implementation of 3D-SRU-Net[25] exists. Thus, we implemented it using Pytorch. The code of our implementation has been uploaded to GitHub (https://github.com/Luchixiang/EMDiffuse/tree/master/3D-SR-Unet). The isotropic volume was cropped into 96 x 96 x 96 subvolumes for training. The initial number of convolution filters was 32. The depth of the model was 3, with each level hosting three convolution layers. Following the paper, the Adam optimizer was employed with a stepwise, square-root learning rate schedule. The initial learning rate was 1e-4. Cubic-weighted PSNR was used as the loss function for training. The network training was stopped when the validation loss didn't decrease for 20 epochs, and the checkpoint with the best performance on the validation set was selected for testing.

CARE 3D reconstruction training: CARE[5] was installed from GitHub (https://github.com/CSBDeep/CSBDeep). For Openorganelle mouse Kidney isotropic reconstruction, the training and inference volumes were both the downsampled 8 nm x 8 nm x 48 nm kidney volume, the same as vEMDiffuse-a. The official anisotropic transform function was employed to downgrade XY view images for training. Unlike its common application on fluorescence microscopy images, the PSF kernel was fixed as 1 due to the small wavelength of electrons. The subsample rate was 6 to meet the 48 nm axial resolution. 18,000 patches were cropped with size 128 x 128 from the anisotropic training volume for training and 2,000 patches were cropped for validation. The depth of the network was 2. The initial learning rate was 4e-4. The MAE was used as the loss function.

Organelle segmentation implementation details

A 3D U-Net model[26] served as the segmentation expert. We trained two separate models for mitochondria and ER segmentation using the bcedice loss function:

$$L = 1 - \frac{A \cap B}{A \cup B} + A \cdot \log B + (1 - A) \cdot \log(1 - B) \tag{19}$$

Where A represents the predicted segmentation mask of the model, and B denotes the GT mask. ∩ symbolizes the intersection of two masks, while ∪ represents the union. The segmentation model was trained using the Adam optimizer with an initial learning rate of 2e-4. We used small

170 × 170 × 80 patches with a stride size of 90 × 90 × 40 for both training and inference due to GPU memory limitations. In the inference stage, we classified pixels with a probability above 0.5 as organelle and the rest as background.

Neurite tracing

Neurite tracing application of vEMDiffuse-a was validated on the MANC dataset[27]. A subvolume of the isotropic dataset (8 nm x 8 nm x 8 nm resolution) was firstly downsampled to an anisotropic volume (8 nm x 8 nm x 48 nm resolution) to train vEMDiffuse-a. The trained vEMDiffuse-a was then applied to another downsampled subvolume of the MANC dataset. To reconstruct neurites from the EM imagery, multi-scale flood-filling networks[28] (FFN) as indicated in the MANC paper[27] was adopted. The FFN model was trained on a 512 x 512 x 512 isotropic volume with the corresponding neurite ground truth mask. The FOV of FFN was (32,32,32), and the depth of the model was 12. The batch size was 4. Then the trained model was applied to ITK-cubic interpolated volume, vEMDiffuse-a generated volume to generate neurite masks. The segmentation results were enhanced through multi-seed over-segmentation, multi-scale over-segmentation and agglomeration[28].

Supplementary References

1. e Drigo, R.A. et al. Age mosaicism across multiple scales in adult tissues. *Cell Metabolism* **30**, 343-351. e343 (2019).
2. Ho, J., Jain, A. & Abbeel, P. Denoising diffusion probabilistic models. *Advances in Neural Information Processing Systems* **33**, 6840-6851 (2020).
3. Ronneberger, O., Fischer, P. & Brox, T. U-net: Convolutional networks for biomedical image segmentation. *Medical Image Computing and Computer-Assisted Intervention*, 234-241 (2015).
4. Saharia, C. et al. Image super-resolution via iterative refinement. *IEEE Transactions on Pattern Analysis and Machine Intelligence* **45**, 4713-4726 (2022).
5. Weigert, M. et al. Content-aware image restoration: pushing the limits of fluorescence microscopy. *Nature Methods* **15**, 1090-1097 (2018).
6. Buchholz, T.-O. et al. Content-aware image restoration for electron microscopy. *Methods in cell biology* **152**, 277-289 (2019).
7. Fang, L. et al. Deep learning-based point-scanning super-resolution imaging. *Nature Methods* **18**, 406-416 (2021).
8. Smith, L.N. A disciplined approach to neural network hyper-parameters: Part 1--learning rate, batch size, momentum, and weight decay. *arXiv preprint arXiv:1803.09820* (2018).
9. Loshchilov, I. & Hutter, F. Stochastic gradient descent with warm restarts. *Proc. of the 5th International Conference Learning Representations*, 1-16 (2017).
10. Zhang, Y. et al. Image super-resolution using very deep residual channel attention networks. *Proc. of the European Conference on Computer Vision*, 286-301 (2018).
11. Kingma, D.P. & Ba, J. Adam: A method for stochastic optimization. *arXiv preprint arXiv:1412.6980* (2014).
12. Lehtinen, J. et al. Noise2Noise: Learning image restoration without clean data. *arXiv preprint arXiv:1803.04189* (2018).
13. Krull, A., Buchholz, T.-O. & Jug, F. Noise2void-learning denoising from single noisy images. *Proc. of the IEEE Conference on Computer Vision and Pattern Recognition*, 2129-2137 (2019).
14. Mittal, A., Moorthy, A.K. & Bovik, A.C. Blind/referenceless image spatial quality evaluator. *2011 conference record of the forty fifth asilomar conference on signals, systems and computers (ASILOMAR)*, 723-727 (2011).
15. Qiao, C. et al. Evaluation and development of deep neural networks for image super-resolution in optical microscopy. *Nature Methods* **18**, 194-202 (2021).
16. Zhang, L., Zhang, L., Mou, X. & Zhang, D. FSIM: A feature similarity index for image quality assessment. *IEEE Transactions on Image Processing* **20**, 2378-2386 (2011).
17. Zhang, R., Isola, P., Efros, A.A., Shechtman, E. & Wang, O. The unreasonable effectiveness of deep features as a perceptual metric. *Proc. of the IEEE Conference on Computer Vision and Pattern Recognition*, 586-595 (2018).
18. Kovesi, P. Image features from phase congruency. *Videre: Journal of computer vision research* **1**, 1-26 (1999).
19. Jain, R., Kasturi, R. & Schunck, B.G. Machine vision, Vol. 5. (McGraw-hill New York, 1995).

20. Sara, U., Akter, M. & Uddin, M.S. Image quality assessment through FSIM, SSIM, MSE and PSNR—a comparative study. *Journal of Computer and Communications* **7**, 8-18 (2019).
21. Wang, X. et al. Esrgan: Enhanced super-resolution generative adversarial networks. *Proc. of the European Conference on Computer Vision (ECCV) Workshops*, 0-0 (2018).
22. Esser, P., Rombach, R. & Ommer, B. Taming transformers for high-resolution image synthesis. *Proc. of the IEEE Conference on Computer Vision and Pattern Recognition*, 12873-12883 (2021).
23. Rombach, R., Blattmann, A., Lorenz, D., Esser, P. & Ommer, B. High-resolution image synthesis with latent diffusion models. *Proc. of the IEEE/CVF Conference on Computer Vision and Pattern Recognition*, 10684-10695 (2022).
24. Descloux, A., Grußmayer, K.S. & Radenovic, A. Parameter-free image resolution estimation based on decorrelation analysis. *Nature Methods* **16**, 918-924 (2019).
25. Heinrich, L., Bogovic, J.A. & Saalfeld, S. Deep learning for isotropic super-resolution from non-isotropic 3D electron microscopy. *Medical Image Computing and Computer-Assisted Intervention*, 135-143 (2017).
26. Heinrich, L. et al. Whole-cell organelle segmentation in volume electron microscopy. *Nature* **599**, 141-146 (2021).
27. Takemura, S.-y. et al. A connectome of the male drosophila ventral nerve cord. *BioRxiv*, 2023.2006. 2005.543757 (2023).
28. Januszewski, M. et al. High-precision automated reconstruction of neurons with flood-filling networks. *Nature Methods* **15**, 605-610 (2018).
