# Supplementary material for: Diffusion-based deep learning method for augmenting ultrastructural imaging and volume electron microscopy
Source: Nat Commun. 2024 Jun 1;15:4677. doi: 10.1038/s41467-024-49125-z (PMC11144272; doi:10.1038/s41467-024-49125-z)
Supplement: Supplementary file 3 — Description of Additional Supplementary Files [file 41467_2024_49125_MOESM3_ESM.pdf]

Description of Additional Supplementary Files

Supplementary Movie 1: Representative illustrative examples of enhanced image quality achieved through EMDiffuse-n denoising on a mouse brain cortex EM image.

Supplementary Movie 2: Comparison of EMDiffuse-n with other denoising methods on an enlarged region of mouse brain cortex EM image. Predictions processed with CARE, PSSR, and RCAN are compared with results obtained with EMDiffuse-n.

Supplementary Movie 3: Transferability of in EMDiffuse denoising. The raw image, image denoised with EMDiffuse-n without fine-tuning, and image denoised with EMDiffuse after fine-tuning are evaluated across datasets from three different tissues: mouse liver, mouse heart, and bone marrow.

Supplementary Movie 4: Representative illustrative examples of super-resolution achieved through EMDiffuse-r on mouse brain cortex EM images.

Supplementary Movie 5: Comparison of EMDiffuse-r with other super-resolution methods on an enlarged region of mouse brain cortex EM image. Results of super-resolution via CARE, PSSR, and RCAN are compared with those achieved through EMDiffuse-r.

Supplementary Movie 6: Examples of transfer learning in EMDiffuse super-resolution. The raw image and image super-resolved with EMDiffuse-r after fine-tuning are evaluated across three distinct tissues: mouse liver, mouse heart, and bone marrow.

Supplementary Movie 7: vEMDiffuse-i restores isotropic volume from downgraded Openorganelle kidney (jrc_mus-kidney) volume. Left: XY views of ultrastructural change within volume reconstructed by vEMDiffuse-i derived from the anisotropic volume with 8 nm x 8 nm x 48 nm voxel size. Right: XY views of ultrastructural change within ground truth isotropic volume with 8 nm x 8 nm x 8 nm voxel size.

Supplementary Movie 8: vEMDiffuse-i restores isotropic volume from downgraded Openorganelle liver (jrc_mus-liver) volume. Left: XY views of ultrastructural change within volume reconstructed by vEMDiffuse-i derived from the anisotropic volume with 8 nm x 8 nm x 48 nm voxel size. Right: XY views of ultrastructural change within ground truth isotropic volume with 8 nm x 8 nm x 8 nm voxel size.

Supplementary Movie 9: 3D reconstruction and rendering of endoplasmic reticulum (ER) and mitochondria within Openorganelle liver volume. Left: ER and mitochondria reconstructions derived from anisotropic volume. Right: ER and mitochondria reconstructions obtained from vEMDiffuse-i restored volume.

Supplementary Movie 10: vEMDiffuse-a restores isotropic volume from downgraded Openorganelle kidney (jrc_mus-kidney) volume without training with isotropic data. Left: XY views of ultrastructural change within volume reconstructed by vEMDiffuse-a derived from the anisotropic volume with 8 nm x 8 nm x 48 nm voxel size. Right: XY views of ultrastructural change within ground truth isotropic volume with 8 nm x 8 nm x 8nm voxel size.

Supplementary Movie 11: vEMDiffuse-a restores isotropic volume from downgraded Openorganelle liver (jrc_mus-liver) volume without training with isotropic data. Left: XY views of ultrastructural change within volume reconstructed by vEMDiffuse-a derived from the anisotropic volume with 8 nm x 8 nm x 48 nm voxel size. Right: XY views of ultrastructural change within ground truth isotropic volume with 8 nm x 8 nm x 8 nm voxel size.

Supplementary Movie 12: Illustrations of YZ views of vEMDiffuse-a enhanced Openorganelle kidney (jrc_mus-kidney) and liver (jrc_mus-liver) volumes.

Supplementary Movie 13: 3D reconstruction and rendering of mitochondria within Openorganelle kidney volume. Yellow: mitochondria reconstructions from anisotropic volume. Red: Organelle reconstruction from vEMDiffuse-a restored volume.

Supplementary Movie 14: vEMDiffuse-a generates isotropic resolution volume from the anisotropic MICrONS multi-area volume. Left: XY views of ultrastructural change within volume generated by vEMDiffuse-a from anisotropic volume. Right: XY views of ultrastructural change in anisotropic volume.

Supplementary Movie 15: vEMDiffuse-a generates isotropic resolution volume from the anisotropic FANC volume. Left: XY views of ultrastructural change within volume generated by vEMDiffuse-a from anisotropic volume. Right: XY views of ultrastructural change in anisotropic volume.

Supplementary Movie 16: Illustrations of YZ views of vEMDiffuse-a generated isotropic FANC and MICrONS multi-area volume.
