# Supplementary material for: Diffusion-based deep learning method for augmenting ultrastructural imaging and volume electron microscopy
Source: Nat Commun. 2024 Jun 1;15:4677. doi: 10.1038/s41467-024-49125-z (PMC11144272; doi:10.1038/s41467-024-49125-z)
Supplement: Supplementary file 20 — Reporting Summary [file 41467_2024_49125_MOESM20_ESM.pdf]

nature portfolio

Corresponding author(s): Haibo Jiang, Xiaojuan Qi

Last updated by author(s): Nov 25, 2023

Reporting Summary

Nature Portfolio wishes to improve the reproducibility of the work that we publish. This form provides structure for consistency and transparency in reporting. For further information on Nature Portfolio policies, see our Editorial Policies and the Editorial Policy Checklist.

Statistics

For all statistical analyses, confirm that the following items are present in the figure legend, table legend, main text, or Methods section.

n/a	Confirmed	
☐	☒	The exact sample size (*n*) for each experimental group/condition, given as a discrete number and unit of measurement
☐	☒	A statement on whether measurements were taken from distinct samples or whether the same sample was measured repeatedly
☐	☒	The statistical test(s) used AND whether they are one- or two-sided<br>*Only common tests should be described solely by name; describe more complex techniques in the Methods section.*
☒	☐	A description of all covariates tested
☐	☒	A description of any assumptions or corrections, such as tests of normality and adjustment for multiple comparisons
☐	☒	A full description of the statistical parameters including central tendency (e.g. means) or other basic estimates (e.g. regression coefficient) AND variation (e.g. standard deviation) or associated estimates of uncertainty (e.g. confidence intervals)
☒	☐	For null hypothesis testing, the test statistic (e.g. F, t, r) with confidence intervals, effect sizes, degrees of freedom and P value noted<br>*Give P values as exact values whenever suitable.*
☒	☐	For Bayesian analysis, information on the choice of priors and Markov chain Monte Carlo settings
☐	☒	For hierarchical and complex designs, identification of the appropriate level for tests and full reporting of outcomes
☒	☐	Estimates of effect sizes (e.g. Cohen's *d*, Pearson's *r*), indicating how they were calculated

Our web collection on statistics for biologists contains articles on many of the points above.

Software and code

Policy information about availability of computer code

Data collection	For vEM, all OpenOrganelle datasets are downloaded from the OpenOrganelle website (https://openorganelle.janelia.org). The Openorganelle Kidney dataset is available at https://doi.org/10.25378/janelia.16913035.v1. The Openorganelle Liver dataset is available at https://doi.org/10.25378/janelia.16913047.v1. The Openorganelle T-Cell dataset is available at https://doi.org/10.6084/m9.figshare.14447541.v1. The EPFL mouse brain dataset is available at https://www.epfl.ch/labs/cvlab/data/data-em/. The MICrONS multi-area dataset can be downloaded from https://www.microns-explorer.org/. The FANC dataset can be downloaded from https://bossdb.org/project/phelps_hildebrand_graham2021. The MANC dataset can be downloaded from https://www.janelia.org/project-team/flyem/manc-connectome. For each dataset, we downloaded a subvolume for training and another different subvolume for test.
Data analysis	The networks for diffusion model were built using PyTorch 1.11. Customized Python3 scripts were built to do registration, image segmentation and statistical evaluation. Fiji and Imaris 9.9 were used for display vEM and rendering the segmentation results. The code is available at https://github.com/Luchixiang/EMDiffuse. The download information was added into the code availability.

For manuscripts utilizing custom algorithms or software that are central to the research but not yet described in published literature, software must be made available to editors and reviewers. We strongly encourage code deposition in a community repository (e.g. GitHub). See the Nature Portfolio guidelines for submitting code & software for further information.

Data

Policy information about availability of data

All manuscripts must include a data availability statement. This statement should provide the following information, where applicable:

- Accession codes, unique identifiers, or web links for publicly available datasets
- A description of any restrictions on data availability
- For clinical datasets or third party data, please ensure that the statement adheres to our policy

Denoising and super-resolution training and test data for EMDiffuse are available at https://zenodo.org/record/8136295. For vEM, all OpenOrganelle datasets are downloaded from the OpenOrganelle website (https://openorganelle.janelia.org). The Openorganelle Kidney dataset is available at https://doi.org/10.25378/janelia.16913035.v1. The Openorganelle Liver dataset is available at https://doi.org/10.25378/janelia.16913047.v1. The Openorganelle T-Cell dataset is available at https://doi.org/10.6084/m9.figshare.14447541.v1. The EPFL mouse brain dataset is available at https://www.epfl.ch/labs/cvlab/data/data-em/. The MICrONS multi-area dataset can be downloaded from https://www.microns-explorer.org/. The FANC dataset can be downloaded from https://bossdb.org/project/phelps_hildebrand_graham2021. The MANC dataset can be downloaded from https://www.janelia.org/project-team/flyem/manc-connectome.

Research involving human participants, their data, or biological material

Policy information about studies with human participants or human data. See also policy information about sex, gender (identity/presentation), and sexual orientation and race, ethnicity and racism.

Reporting on sex and gender	*Use the terms sex (biological attribute) and gender (shaped by social and cultural circumstances) carefully in order to avoid confusing both terms. Indicate if findings apply to only one sex or gender; describe whether sex and gender were considered in study design; whether sex and/or gender was determined based on self-reporting or assigned and methods used.*<br>*Provide in the source data disaggregated sex and gender data, where this information has been collected, and if consent has been obtained for sharing of individual-level data; provide overall numbers in this Reporting Summary. Please state if this information has not been collected.*<br>*Report sex- and gender-based analyses where performed, justify reasons for lack of sex- and gender-based analysis.*
Reporting on race, ethnicity, or other socially relevant groupings	*Please specify the socially constructed or socially relevant categorization variable(s) used in your manuscript and explain why they were used. Please note that such variables should not be used as proxies for other socially constructed/relevant variables (for example, race or ethnicity should not be used as a proxy for socioeconomic status).*<br>*Provide clear definitions of the relevant terms used, how they were provided (by the participants/respondents, the researchers, or third parties), and the method(s) used to classify people into the different categories (e.g. self-report, census or administrative data, social media data, etc.)*<br>*Please provide details about how you controlled for confounding variables in your analyses.*
Population characteristics	*Describe the covariate-relevant population characteristics of the human research participants (e.g. age, genotypic information, past and current diagnosis and treatment categories). If you filled out the behavioural & social sciences study design questions and have nothing to add here, write "See above."*
Recruitment	*Describe how participants were recruited. Outline any potential self-selection bias or other biases that may be present and how these are likely to impact results.*
Ethics oversight	*Identify the organization(s) that approved the study protocol.*

Note that full information on the approval of the study protocol must also be provided in the manuscript.

Field-specific reporting

Please select the one below that is the best fit for your research. If you are not sure, read the appropriate sections before making your selection.

☒ Life sciences ☐ Behavioural & social sciences ☐ Ecological, evolutionary & environmental sciences

For a reference copy of the document with all sections, see nature.com/documents/nr-reporting-summary-flat.pdf

Life sciences study design

All studies must disclose on these points even when the disclosure is negative.

Sample size	The sample size of each experiment is provided in the figure legends in the main manuscript and supplementary information files. And we performed at least 3 replications to ensure reproducibility.
Data exclusions	No data were excluded from data analysis.
Replication	All experiments were repeated at least 3 times. For denoising and super-resolution, we repeated the experiments for at least 10 regions. For vEM reconstruction, we repeated on experiments at least 3 times for each dataset and no failed reconstruction was observed.

Randomization	The samples were randomly chosen before imaging experiments. Training and testing datasets for the network model were randomly generated. Validation data was randomly chosen from the experimental dataset.
Blinding	All the performance test of the trained networks were blindly operated on data that were not included in the network training process.

Reporting for specific materials, systems and methods

We require information from authors about some types of materials, experimental systems and methods used in many studies. Here, indicate whether each material, system or method listed is relevant to your study. If you are not sure if a list item applies to your research, read the appropriate section before selecting a response.

Materials & experimental systems

n/a	Involved in the study	
☒	☐	Antibodies
☐	☒	Eukaryotic cell lines
☒	☐	Palaeontology and archaeology
☐	☒	Animals and other organisms
☒	☐	Clinical data
☒	☐	Dual use research of concern
☒	☐	Plants

Methods

n/a	Involved in the study	
☒	☐	ChIP-seq
☒	☐	Flow cytometry
☒	☐	MRI-based neuroimaging

Eukaryotic cell lines

Policy information about cell lines and Sex and Gender in Research

Cell line source(s)	HeLa cells were acquired commercially from the ATCC.
Authentication	The cells were regularly monitored on morphology and proliferation rate.
Mycoplasma contamination	Tested negative for mycoplasma contamination.
Commonly misidentified lines (See ICLAC register)	No commonly misidentified cell lines were used.

Animals and other research organisms

Policy information about studies involving animals; ARRIVE guidelines recommended for reporting animal research, and Sex and Gender in Research

Laboratory animals	C57BL/6 mice (Male, 10~12-week-old) were provided by Animal Resources Centre (Australia), and the procedures were approved by the Animal Ethics Committee of The University of Western Australia.
Wild animals	Not applicable
Reporting on sex	Not applicable
Field-collected samples	Not applicable
Ethics oversight	Procedures were approved by the Animal Ethics Committee of The University of Western Australia.

Note that full information on the approval of the study protocol must also be provided in the manuscript.
